# Perspective Approaches to “Trojan Horse” Strategy Development for Combating Bacterial Pathogens

**DOI:** 10.3390/ph19050701

**Published:** 2026-04-29

**Authors:** Margarita Shleeva, Nataliya Kozobkova, Galina Demina, Arseny Kaprelyants

**Affiliations:** A.N. Bach Institute of Biochemistry, Federal Research Centre ‘Fundamentals of Biotechnology’, Russian Academy of Sciences, 119071 Moscow, Russia; natalia.cosolapowa@gmail.com (N.K.); galyademina@yandex.ru (G.D.);

**Keywords:** targeted drug delivery, Trojan Horse strategy, bacterial transporters, drug conjugates

## Abstract

**Background/Objectives:** The escalating crisis of antibiotic resistance and the inherent limitations of conventional antibiotics necessitate the development of innovative therapeutic strategies. Targeted drug delivery (TDD) offers a powerful approach to enhance efficacy, minimize systemic toxicity, and circumvent bacterial resistance. This systematic review aims to evaluate the potential of unique bacterial transport systems (BTSs), surface specific receptors and intracellular enzymes as platforms for TDD via the “Trojan Horse” strategy (THS). **Methods:** A comprehensive literature review was conducted, focusing on studies that investigated the specificity and mechanisms of BTSs responsible for the uptake of metabolites that are essential for and unique to bacteria. This includes an analysis of transport systems for siderophores, bacteria-specific sugars, cell wall components, D-amino acids, and vitamins. We assessed preclinical and clinical examples of drug conjugates utilizing these pathways, as well as emerging platforms such as bacteriophage-derived proteins, antibody–antibiotic conjugates, and bacterial extracellular vesicles (EVs). **Results:** BTSs demonstrate high specificity for their cognate substrates, providing effective molecular gateways for TDD of drugs photosensitizers and diagnostic probes in form of conjugates. The siderophore–cephalosporin conjugate cefiderocol represents a clinically validated example, having received FDA approval. Preclinical studies further reveal that conjugates utilizing sugars (e.g., maltose, trehalose) and vitamins (e.g., B12) can significantly enhance antibiotic uptake and activity against both Gram-positive and Gram-negative pathogens, including drug-resistant strains. Emerging platforms like bacteriophage endolysins and engineered EVs show promise for overcoming biological barriers such as bacterial outer membranes and intracellular host niches. **Conclusions:** The THS leveraging BTSs represents a clinically viable and promising avenue for next-generation antibacterial therapies. Advantages of BTS include overcoming bacterial resistance, such as reduced membrane permeability and efflux pumps, enabling the “revival” of antibiotics that are poorly permeable or toxic, increasing their local concentration at the target site and reducing side effects on host cells. While significant progress has been made, a striking disconnect persists between the hundreds of conjugates demonstrating potent in vitro activity and the limited agent that has achieved clinical use. This in vitro–in vivo gap reflects, in large part, the early stage of this field rather than a fundamental failure. Further research is critically needed not only to identify novel BTSs and optimize drug-linker chemistry, but also to systematically address the translational barriers—including poor pharmacokinetics, immunogenicity, and unexpected toxicity—that have prevented most promising candidates from advancing beyond preclinical evaluation.

## 1. Introduction

The escalating crisis of antibiotic resistance, together with the intrinsic shortcomings of traditional antimicrobials, poses an existential threat to modern medicine [1]. The core problem is not just the misuse of antibiotics, but also their ineffectiveness against already resistant strains. Furthermore, even when antibiotics are effective, their broad-spectrum action leads to negative consequences. These include disruption of the microbiome, the development of secondary infections, and the emergence of side effects [2].

Traditional approaches to drug delivery often rely on systemic administration. However, they face significant challenges, such as systemic toxicity, poor bioavailability, and off-target effects on healthy tissues. These limitations reduce therapeutic efficacy and increase the risk of adverse events. Such issues are particularly acute in the treatment of infectious diseases, where achieving sufficiently high antibiotic concentrations at the infection site is crucial. This is necessary both to overcome pathogen resistance and prevent its further development [3]. Inadequate drug concentrations in the targeted area allow a subpopulation of pathogens to survive. This incomplete exposure fails to resolve the infection and facilitates the spread of the surviving organisms. Critically, it also promotes the selection and propagation of resistant strains due to the sustained, low-level drug exposure [4]. Therefore, there is increasing interest in using biological systems for targeted drug delivery (TDD). Bacteria are recognized as a particularly promising approach, especially for fighting infectious diseases [5]. This strategy maximizes drug efficacy, minimizes systemic side effects, and reduces the likelihood of resistance development by avoiding unnecessary exposure of non-target microbes [6]. Targeted delivery improves antibiotic concentration at infection sites, overcoming barriers such as bacterial biofilms and intracellular localization, which often render traditional drugs ineffective [7,8].

To date, several approaches have been proposed for the implementing TDD [3]. These include the use of various stimuli-responsive nanocarriers [9,10,11], and pH-responsive drug delivery systems [9].

At the same time, bacteria possess unique characteristics that provide the basis for TDD in their own right. In particular, bacteria have remarkable transport systems. These are complex molecular mechanisms that efficiently move substances across cellular envelopes. These bacterial transport systems (BTSs) are critical for maintaining cellular homeostasis. They enabling the uptake of vital nutrients—some of which are unique to bacteria—from the environment, as well as the excretion of metabolites. The existence of such transport systems creates the prerequisites for the developing conjugates containing a naturally transported substance with an antibiotic compound. Once transported into the bacterial cell, the antibiotic exerts its detrimental effect.

Furthermore, the bacterial cell is surrounded by unique structures, such as the cell wall and capsule. These structures create an opportunity for specific binding. Molecules and supramolecular structures with high affinity for these cellular components can be conjugated with antibiotic agents or other target molecules. These two approaches form the basis for the “Trojan Horse” Strategy (THS), which is one of the promising modalities for implementing TDD. The present review examines the THS with an emphasis on BTSs for key metabolites (sugars, peptidoglycan fragments, siderophores, D-amino acids, vitamins), surface-binding platforms (antibodies, lectins, phages), and emerging systems (extracellular vesicles (EVs), prodrugs). We critically evaluate the advantages, limitations, and translational challenges of each approach. We discuss strategies for creating conjugates of these ligands with therapeutic agents and highlight preclinical examples that demonstrate improved bioavailability, reduced toxicity, and enhanced efficacy. In parallel, we examine bacteriophage-derived proteins and surface receptors-based structures as an emerging platform for targeted delivery. These function as a type of THS by leveraging their inherent specificity for bacterial recognition and their capacity to deliver diverse therapeutic payloads, including antibiotics and CRISPR-Cas systems. Furthermore, we critically evaluate the advantages and limitations of each platform, identify persistent translational challenges, and propose concrete research priorities for the next decade.

## 2. The “Trojan Horse” Strategy in Drug Delivery

The THS uses a carrier molecule—designed to mimic a bacterial nutrient, ligand, or surface-binding entity—to deliver a therapeutic payload (antibiotic, photosensitizer, or probe) into or onto target bacteria. By exploiting bacterial recognition systems, the THS bypasses passive defenses such as impermeable membranes and efflux pumps.

Critically, this approach bypasses many of the natural defenses that bacteria employ against external threats, including antibiotics. These defenses include impermeable cell walls/membranes, efflux pumps, and enzymatic degradation [12]. The “Trojan Horse” is recognized as a “friendly” and necessary molecule. As a result, it bypasses these passive and active exclusion mechanisms [13,14].

Thus, the THS offers promise for overcoming bacterial resistance linked to reduced drug permeability and efflux pumps. It enables targeted delivery of cargo to pathogenic bacteria. This approach also holds the potential for enhanced antimicrobial activity and reduced side effects.

In many publications, the term THS is used in connection with siderophores conjugated with various substances as carriers (see below). Historically, these represent the first examples of this strategy. However, in our view, the THS can be expanded to include other naturally transported compounds. These include sugars, amino acids, peptides, and others, as discussed later in this review. Furthermore, within the THS we also include compounds or structures that specifically bind to the bacterial cell surface as carriers. Examples include antibodies or phage structures. In this case, cell-surface receptors function as bait for antibiotic-loaded carriers, thus representing a component of the THS. As a result, this strategy minimizes exposure of host cells to the drug, which potentially reduces side effects. The high intracellular concentration of the released drug can lead to more potent antimicrobial activity, even against less susceptible bacteria [14,15,16]. Furthermore, this enables the transport of substances into the pathogen cell. Specifically, antibiotics that do not normally penetrate the cell for various reasons—yet have intracellular targets—can be delivered effectively.

The main principles of the THS include the following: (a) the carrier should be a “native” substance for the pathogen and should not be toxic to the cell *per se*. This property should significantly reduce the development of resistance to the conjugate as a whole. Particularly advantageous is the use of a carrier (e.g., a substrate) that is essential for bacterial survival. This should further diminish the emergence of resistance. (b) Ideally, the carrier or cell-binding compounds or structures must be unique to the pathogen. They should not be transported by or bound to host cells. This provides the basis for specificity.

### 2.1. Mechanism of “Trojan Horse” Strategy Action

The unifying principle across all Trojan Horse modalities, whether based on low-molecular-weight metabolites or supramolecular carriers, is the exploitation of a bacterium’s own recognition systems. In each case, the carrier is designed or selected to be recognized by the target bacterium—either as a substrate for essential nutrient transporters (e.g., siderophores, sugars, vitamins) or as a ligand for surface structures (e.g., lectins, teichoic acids, phage receptors). This recognition enables targeted delivery, bypassing passive diffusion barriers and minimizing off-target effects. Thus, while the physical nature of the carrier varies, all approaches share the core conceptual framework of using bacterial-specific interactions to deliver a therapeutic payload.

Bacteria have evolved sophisticated systems to acquire vital resources from their environment. These essential molecules often include: (1) nutrients (such as iron, sugars, or amino acids)—which are critical for bacterial metabolism and replication [17]; (2) growth factors (specific vitamins or cofactors) that are indispensable for bacterial processes [18]; (3) siderophores—small, high-affinity iron-chelating molecules secreted by many bacteria to scavenge scarce iron from their surroundings [19]; and (4) other specific ligands—molecules that bind to unique bacterial surface receptors, which might be involved in nutrient uptake, adhesion, or even entry into host cells (in the case of intracellular pathogens) [20].

The bacterial cell actively recognizes and binds to the “Trojan Horse” complex. This recognition is driven by its inherent need for the essential molecule that the carrier mimics. This binding typically occurs via specific surface receptors or transport proteins that are dedicated to importing the natural nutrient or molecule.

Once the “Trojan Horse” complex has traversed the bacterial cell envelope and entered the cytoplasm or periplasm, a second crucial step occurs: the release of the therapeutic agent. The release mechanism is often triggered by the intracellular environment of the bacterium. This can include: pH changes [9] (the internal pH of a bacterium might differ from the external environment, cleaving the bond between the carrier and the drug); enzymatic activity (intracellular bacterial enzymes can be designed to specifically break down the linker molecule holding the drug to the carrier); and redox potential (differences in oxidation–reduction potential within the cell can also be used to trigger drug release) [10]. As a result, the therapeutic agent is localized precisely inside the target bacterium, either in its released active form or as an integral part of the conjugate. Its effective concentration can therefore be significantly higher than if it had been administered conventionally.

When supramolecular structures such as antibodies or phage-derived particles are employed as carriers, the release or delivery of the active agent follows mechanisms that are distinct from those of low-molecular-weight conjugates. If the supramolecular carrier (e.g., an antibody–antibiotic conjugate, AAC, or a phage-based nanocarrier) is designed to be internalized by host cells (e.g., macrophages) or by the bacteria themselves, the active agent is typically released upon degradation of the carrier. In AACs, for instance, the linker connecting the antibiotic to the antibody is cleaved by proteolytic enzymes (such as cathepsins) inside the host cell. This liberates the free, active antibiotic to target intracellular pathogens [14,21,22].

In many applications, however, the primary goal is not internalization into the bacterium. Instead, it is the high-precision delivery of a toxic payload directly to the bacterial cell surface. In this scenario, the active agent acts while still conjugated to the supramolecular carrier [23,24].

In this regard, photosensitizers or photothermal agents can be conjugated to antibodies or phages. Upon light activation, they generate lethal reactive oxygen species or heat. This destroys the bacterial membrane without needing to be released from the carrier [25,26,27].

Carrier-bound antimicrobial peptides can disrupt the bacterial membrane integrity from the surface [28,29].

Some supramolecular carriers, such as hydrogels containing functionalized cellulose [30,31] or extracellular vesicles [32,33], can act as localized drug depots.

In these and other examples the active agent is released extracellularly in the immediate vicinity of the bacteria. This release can be passive, through gradual diffusion from the carrier matrix [30,34], or triggered by specific signals in the infection microenvironment, such as bacterial enzymes (e.g., β-lactamases, proteases) [35,36], pH changes [9], or hypoxia [11], leading to carrier disassembly and subsequent drug release.

Thus, in the context of supramolecular carriers, the active agent can be deployed either on-site (acting directly from the carrier) or in-site (released intra- or extracellularly) to exert its bactericidal effect.

As a result, by specifically targeting bacterial uptake or binding systems, this strategy minimizes exposure of host cells to the drug, potentially reducing side effects. The high intracellular concentration of the released drug can lead to more potent antimicrobial activity, even against less susceptible bacteria [14,15,16].

Essentially, the THS represents an approach that exploits multiple vulnerabilities of the bacterial cell, associated with the presence of transport systems, specific cell-surface receptors, and certain intracellular enzymes that are indispensable for bacterial physiology, yet can simultaneously be leveraged against the cell itself. The detailed design principles governing linker selection, activation mechanisms, and pharmacokinetic optimization are discussed in Section 3.

### 2.2. Types of “Trojan Horses”

**Figure 1:**
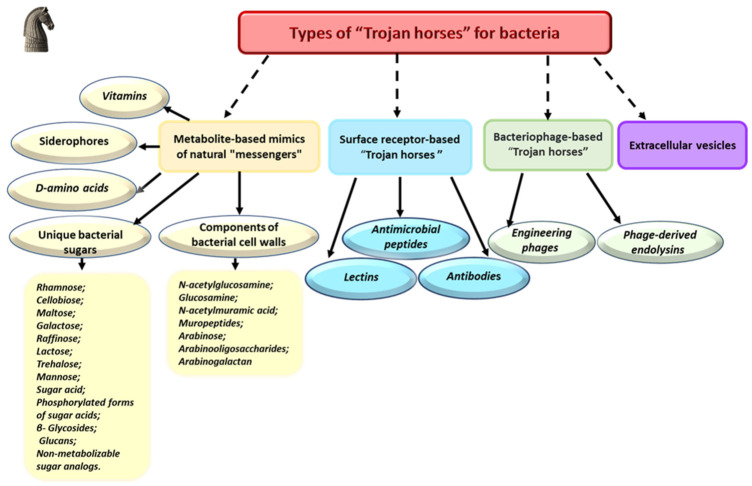
Classification of bacterial “Trojan Horse” delivery systems based on their mechanisms of action. The colors reflect the mechanism of THS interaction with target bacteria: yellow—active transport; blue—receptor binding; green—specific adhesion; purple—fusion/depot release. Dashed arrows—“Trojan Horse” mechanisms; solid arrows—specific carriers ("horses") within each mechanism.

Classification of “Trojan Horses” by delivery mechanism allows a better understanding of their mode of action and potential for clinical applications. The main types of such systems grouped according to their mechanism and mode of entry into the bacterial cell are shown on Figure 1. The classification presented in Figure 1 distinguishes between two broad implementation strategies for the Trojan Horse concept. The first relies on metabolite-based mimics, which are small molecules (e.g., siderophores, sugars, D-amino acids, vitamins) that are actively transported into the bacterial cell via dedicated uptake systems [37]. Here, the carrier itself serves as a “molecular key” that grants the conjugated antibiotic access to the cytoplasm or periplasm. The second comprises surface receptor-based systems, which include larger biological entities such as antibodies, lectins, bacteriophages, and extracellular vesicles. These platforms function either by binding to bacterial surface receptors to deliver a payload directly to the cell envelope (e.g., photosensitizers, lytic enzymes) or by exploiting host cell uptake mechanisms (e.g., phagocytosis) to release the drug inside intracellular compartments where pathogens reside.

#### 2.2.1. Metabolite-Based Mimics of Natural “Messengers” and Transport Mechanisms

These “Trojan Horses” mimic molecules or processes naturally used by bacteria to acquire essential resources or transport substances. The bacterial world is remarkable not only for its diversity but also for its extraordinary ability to adapt to extreme environments. A key element of this adaptation is an efficient system for exchanging metabolites with the surrounding environment, mediated by complex transport systems. While ubiquitous transport mechanisms handle the influx of essential nutrients, certain metabolites are selectively transported by bacteria through dedicated, exclusive systems [20]. These specialized systems not only reflect the unique metabolic requirements of specific bacterial species or communities but also often determine their interactions, survival, and pathogenic potential [20]. Bacteria are equipped with a diverse and intricate array of transport systems that mediate the uptake of necessary nutrients and the removal of toxic compounds. Notably, some of these systems are unique to specific bacterial species or groups, rendering them promising platforms for TDD via the THS [20,38].

Key characteristics of BTSs that make them attractive for the THS include high specificity and efficient transport of substances across the bacterial cell wall and membrane. BTSs are usually targeted at specific metabolites or signaling molecules, which ensures selective transport into bacterial target cells [39].

Unique BTSs may be classified according to the type of molecules they transport and their mechanism of action.

##### Siderophores

Bacteria utilize siderophores to capture iron. Iron is a critically important element for most living organisms, including bacteria. It participates in essential processes such as respiration, DNA synthesis, and antioxidant defense. However, in aerobic environments, iron often exists in poorly soluble Fe(III) forms. This makes it difficult for bacteria to access. In response, bacteria have evolved complex strategies to acquire iron based on the secretion of siderophores.

**Figure 2:**
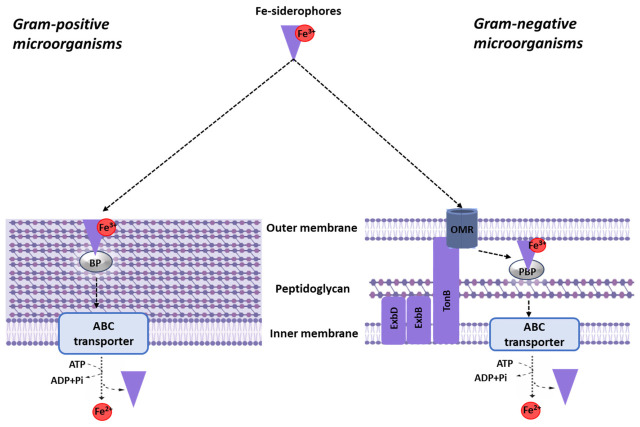
Schematic of siderophore-mediated iron uptake in Gram-positive and Gram-negative bacteria. OMR—outer membrane receptor; TonB, ExbB, ExbD—component of Ton complex; PBP—periplasmic binding protein; BP-binding protein (receptor).

Siderophores are low-molecular-weight compounds (Mw 200–2000 Da) secreted by bacteria, fungi, and plants. They are highly specific chelators of trivalent iron salts. They exhibit marked selectivity and strong binding capability for iron (K_aff_ > 10^30^) [16]. These compounds bind to iron, which is then transported into the cell. This process fulfills the iron requirements of metabolism under conditions of low environmental iron concentration. Based on their chemical structure, siderophores (over 500 compounds) are divided into several classes. This classification depends on the presence of five iron-binding motifs: catecholate, hydroxamate, phenolate, carboxylate, and α-hydroxy carboxylate [40]. Their structure and synthesis by producer cells are well studied (for a review, see, for example, [41]). Siderophores are synthesized through two principal mechanisms. One involves the nonribosomal peptide synthetase (NRPS) pathway. The other is the nonribosomal independent synthesis (NIS) pathway [41]. The transport of iron-complexed siderophores into the bacterial cell differs between Gram-positive and Gram-negative bacteria. In Gram-negative bacteria, the siderophore–iron complex interacts with a receptor on the cell surface, localized in the outer membrane. Active transport of the iron–siderophore complex across the outer membrane is an energy-dependent process [42,43,44] (Figure 2).

Transport of iron–siderophore complexes across the bacterial cell envelope requires energy. In Gram-negative bacteria, the TonB complex harnesses the proton motive force to drive translocation across the outer membrane, while ABC (ATP/adenosine triphosphate-binding cassette) transporters provide energy for import across the cytoplasmic membrane [45,46,47,48,49]. Gram-positive bacteria lack an outer membrane and rely primarily on ABC transporters for siderophore import [43,50]. Critically, the dependence on specific transporters (e.g., TonB-dependent receptors, ABC transporters) makes these systems both highly selective and vulnerable—a feature that has been exploited for targeted drug delivery, though it also represents a potential route for resistance development via transporter mutations [47,51].

The most advanced direction in the use of bacteria-specific transported compounds as conjugates (THS) is currently the siderophore-based approach [52].

In an interesting twist, certain bacteria have evolved to exploit this siderophore-mediated iron acquisition for antibiotic delivery, conjugating toxic compounds to siderophores. These naturally occurring siderophore–drug conjugates, also known as sideromycins [53], represent a promising class of antibiotics. Examples of these sideromycins include the danomycins, salmycins [54], albomycins [55,56], ferrimycins [57,58,59], and microcins [60].

To date, a significant number of different siderophore and antibiotic compound conjugates have been synthesized and tested (for review see [15,16,40,61,62,63,64,65,66,67]). A review [40] lists over 50 such conjugates, including those with beta-lactam antibiotics, fluoroquinolones, cephalosporins, and others, linked to various siderophores via both cleavable and non-cleavable linkers. Most of these demonstrate enhanced efficacy against targeted Gram-positive and Gram-negative bacteria compared to their unconjugated antibiotic counterparts. In some cases, the effectiveness of these conjugates against resistant strains has been demonstrated, particularly against *Pseudomonas aeruginosa*, which exhibits high resistance to a wide range of antibiotics [16]. However, it is important to note that resistance can also develop to siderophore–antibiotic conjugates, as their transport depends on the presence of specific transporters (e.g., TonB-dependent receptors). Mutations that disrupt or reduce the function of these uptake systems represent a primary route of resistance. For instance, laboratory evolution studies have identified mutations in the TonB-dependent siderophore receptor PirA and the ferric enterobactin transporter PiuA that significantly reduce cefiderocol uptake in *P. aeruginosa* [47,51]. Similarly, resistance to the natural sideromycin albomycin arises via loss-of-function mutations in its cognate transporter FhuA [47]. Clinically, while cefiderocol remains highly effective, cases of resistance emergence during therapy have been documented, particularly in patients with prior exposure to siderophore–cephalosporins. Importantly, the fitness cost associated with such transporter mutations varies: mutations in non-essential transporters may be readily tolerated, whereas targeting essential iron-acquisition systems required for growth in vivo may impose a fitness penalty, potentially slowing the spread of resistance. Nevertheless, the vulnerability to transporter mutations is an inherent limitation of any uptake-mediated THS and must be carefully considered during conjugate design [47,51].

For effective antibacterial action, conjugates transported into the cell must undergo intracellular chemical modification to release the inhibitor. This requirement applies when the bacterial target is located in the cytoplasm, with rare exceptions. This is precisely why the structure of the linker is important for the overall activity of the conjugate. The linker connects the siderophore molecule and the antibiotic [53]. Many compounds have been used as linkers. These include amino acids [68], succinic acid [69], alkyl chains [70], polyethylene glycol (PEG) chains [71], triazoles [72], acetal groups [73], disulfide bonds [74], thiol-maleimides [75], citric acid [76], and esters [77]. Among these linkers, some are cleavable within cells and some are non-cleavable. Cleavage occurs through hydrolysis, enzymatic cleavage, and reduction [15,40,64]. However, such linkers can be labile. They may be hydrolyzed too early during intracellular transport or within the host, leading to a loss of activity. An interesting approach involved using a cephalosporin-based linker. This linker is less labile and is selectively cleaved by bacterial β-lactamase [78]. At the same time, conjugates with non-cleavable linkers are effective when the target is localized in the periplasm of Gram-negative bacteria [40].

Siderophores can also mediate the transport of metals that are toxic to bacterial cells and can compete with iron. For instance, a linker-free preparation based on a natural sideromycin complexed with colloidal bismuth citrate has demonstrated significant efficacy against *P. aeruginosa* and *Burkholderia cepacia* in both in vitro and in vivo experiments [79].

Although the majority of studies on siderophore conjugates as antibacterial agents have been conducted in vitro, their efficacy has also been demonstrated in vivo using laboratory models of infection. Several pharmaceutical companies (Pfizer, GSK, Takeda, Synphar, and others) have developed conjugates for clinical trials. These include MC-1, a siderophore-conjugated monocarbam with activity against MDR *P. aeruginosa* and ESBL (extended-spectrum beta-lactamases)-producing members of the *Enterobacteriaceae*. Other examples are BAL30072 from Basilea, a siderophore monosulfactam, and S-649266 from Shionogi Pharmaceuticals, a catechol–cephalosporin antibiotic [66]. Unfortunately, almost all of these were halted at the preclinical stage or Phase I clinical trial. The reasons included instability in the body and side effects. However, a recently developed synthetic catecholate—siderophore–cephalosporin conjugate (Cefiderocol) has received approval from the US Food and Drug Administration (FDA) [40]. This conjugate, developed by Shionogi & Company Ltd., demonstrates activity against a range of carbapenem-resistant Gram-negative pathogens [65]. Siderophores are utilized not only for transporting antibacterial compounds but also, as reported, for delivering photoactivators to enhance the photodynamic inactivation of *P. aeruginosa* [80] and as conjugated vaccine components targeting uropathogenic *E. coli* [81].

##### Unique Bacterial Sugars and Their Dedicated Transport Systems

Sugar metabolism is fundamental to bacterial life. It provides essential building blocks and energy for growth and survival [82]. While many sugars are universally utilized across life forms, bacteria possess unique metabolic pathways and utilize distinct sugar molecules not commonly found or processed in mammals [83,84]. These bacteria-specific sugars, and the dedicated transport systems responsible for their uptake, represent promising targets for the development of the THS.

A key feature of bacterial sugar uptake is the phosphotransferase system (PTS), a unique transport and phosphorylation pathway that is not found in eukaryotic cells [85]. While the PTS is particularly prevalent in Gram-positive bacteria, it is also present in many Gram-negative species [86]. The PTS couples sugar import with phosphorylation, using phosphoenolpyruvate (PEP) as the energy source [85,87,88]. This coupling ensures efficient sugar utilization and is absent in mammalian cells, making PTS-dependent sugars attractive for TDD [87,89]. While many sugars are transported by the PTS, the most promising vectors for the THS are those that are either uniquely bacterial or are not efficiently utilized by mammalian cells—including rhamnose, trehalose, and certain disaccharides discussed below.

Rhamnose

The mechanisms of rhamnose transport into the bacterial cell differ among various microbial species. In *Escherichia coli* and other *Enterobacteriaceae*, the transport of L-rhamnose occurs via a proton-coupled symport system, which is induced when bacteria grow on rhamnose and creates an alkaline pH shift upon adding the sugar to energy-depleted cells [90]. This transport is respiration-dependent and optimal at pH 7. It is inhibited by protonophores and ionophores, confirming its energy-dependent nature [90]. In *Rhizobium leguminosarum*, rhamnose transport is mediated by an ABC transport system encoded by a specialized genetic locus that also contains genes for the catabolism of this sugar [91]. This locus includes genes encoding a putative dehydrogenase, isomerase, and sugar kinase. These enzymes are necessary for the subsequent metabolism of rhamnose. The regulation of these genes is controlled by a DeoR-type repressor (RhaR), which is encoded in the same transcript as the ABC transporter. Expression is induced by the presence of rhamnose in the medium [91]. Alternative rhamnose transport pathways have been found in *Arthrobacter pyridinolis.* These include a PTS and malate- or succinate-dependent transport. The latter stimulates rhamnose uptake in membrane vesicles [92].

Currently, there are several experimental studies where rhamnose or its derivatives have been used to implement the THS (summarized in Table 1).

Thus, a study showed that replacing the native sugar D-desosamine in the macrolide antibiotic YC-17 with L-rhamnose led to improved antibacterial activity against susceptible and resistant strains of *Enterococcus faecium* and *Staphylococcus aureus* compared to the parent compound [93]. New non-azole antifungal and antimicrobial agents were created that use rhamnose esterified with fatty acids to deliver a lethal lipophilic payload to the target within the pathogen’s cell [29].

A novel approach to combat *P. aeruginosa* biofilms has been described using a recombinant protein rHPLOE that specifically binds rhamnose [94], a component of key structures in the *P. aeruginosa* biofilm. The binding of the protein led to a significant reduction in the amount of di-rhamnolipid within the biofilm resulted in inhibition of the formation of a new biofilm and also caused the dispersion (disruption) of an already mature biofilm. This, in turn, enhanced the efficacy of traditional antibiotics, which could now better penetrate the bacterial cells. This study illustrates a THS variant, where rhamnose does not deliver the cargo, but rather the rhamnose-binding protein delivers itself, using bacterial rhamnose as the target (“anchor”) [94].

In addition to rhamnose, in the context of drug delivery, it is also necessary to focus on rhamnolipids (Table 1).

**Table 1:** Representative rhamnose-based Trojan Horse systems.

Platform	Delivery Mechanism	Target Pathogens	Key Outcome
L Rhamnosyl 10 deoxymethyloside (macrolide conjugate)	Direct antibiotic conjugation; hijacks rhamnose transport	*E. faecium*, *S. aureus* (including erythromycin resistant strains)	Improved activity compared to parent compound [93]
Rhamnolipid-functionalized liposomes with cinnamaldehyde	Membrane-mediated delivery via rhamnose recognition	*S. typhimurium*, *S. enteritidis*	Enhanced binding to bacterial membrane; improved antibacterial activity [95]
Rhamnolipid–arginine derivatives (RLmix Arg, monoRL Arg)	Cationic amphiphiles; rhamnose ensures biocompatibility	MRSA, other Gram-positive bacteria	Membrane disruption; enhanced antibacterial activity [28]
Rhamnose-binding protein (rHPLOE)	Lectin-based targeting; binds rhamnose in biofilm matrix	*P. aeruginosa* biofilms	Disrupts biofilms; enhances antibiotic penetration [94]
Rhamnopyranoside-based fatty acid esters	Amphiphilic sugar esters; rhamnose as hydrophilic carrier	*B. subtilis*, *S. aureus*, *E. coli*, *P. aeruginosa*	Broad spectrum antimicrobial activity via membrane penetration [29]

Rhamnolipids are glycolipids produced by *P. aeruginosa* (and other Gram-negative bacteria). They consist of one or two molecules of rhamnose linked to hydroxy-fatty acids. Rhamnolipids are involved in bacterial motility, biofilm formation, and the uptake of hydrophobic substrates [96]. Research indicates that mono-rhamnolipids are well-recognized by bacteria and can be taken up by cells and converted into di-rhamnolipids. This opens possibilities for the delivery of therapeutic agents conjugated with rhamnolipids [96]. Recent developments include the creation of rhamnose liposomes functionalized with rhamnolipids and loaded with cinnamaldehyde. These liposomes demonstrated enhanced antibacterial activity against *Salmonella* due to better binding to the bacterial membrane [95]. By chemically modifying rhamnolipids (by attaching amino acids and, potentially, other cargo), the authors create compounds that likely retain the ability to interact with bacterial membranes or target cells. Rhamnose itself serves as that “familiar” element that facilitates contact [28]. The modified rhamnolipids themselves (especially the arginine derivatives) exhibit enhanced antibacterial activity. The mechanism of action involves the electrostatic interaction of the cationic “head” with the negatively charged bacterial membrane, leading to its disruption. Rhamnose here acts as part of the “Trojan Horse,” ensuring biocompatibility and biodegradability [28].

Summarizing, rhamnose is an ideal candidate for the development of “Trojan Horse” antimicrobial agents. This is due to its unique biochemical role and transport specificity. Confirmed strategies range from direct conjugation with antibiotics to the creation of complex nanoscale platforms (rhamnosomes [95]) and highly specific proteins (lectins [94]) targeting pathogen rhamnose structures. These approaches not only enhance the effectiveness of antimicrobial action but also open pathways for overcoming drug resistance by targeting biofilms and exploiting mechanisms to which bacteria develop resistance more slowly.

##### Cellobiose

Cellobiose, a disaccharide linked by β-1,4-glycosidic bonds, serves as a natural substrate specific for bacterial transport systems, making it a valuable vector for targeted antimicrobial delivery (Table 2). Many pathogenic bacteria, including *Escherichia coli*, *P. aeruginosa*, *Salmonella enterica*, *Klebsiella pneumoniae*, *Clostridium perfringens*, and *Clostridioides difficile,* utilize ABC transporters for cellobiose uptake [97,98]. These systems consist of periplasmic binding proteins, transmembrane channels, and ATPases [99] that actively transport cellobiose into the cytoplasm, where β-glucosidases hydrolyze it to glucose [97,100]. In Gram-negative bacteria, porins facilitate outer membrane passage prior to ABC-mediated import [101]. Regulation involves induction by cellobiose and catabolite repression by glucose, ensuring selective expression under nutrient stress relevant to infections [97,98,102].

The key advantage of cellobiose for the THS lies in its unique β-1,4-linkage, which is specifically cleaved by bacterial β-glucosidases—enzymes absent in mammalian cells. This property enables the design of prodrugs that release active antibiotics only after intracellular hydrolysis, minimizing off-target effects and overcoming permeability-based resistance mechanisms. As an example, cellobiose–nitrofurantoin/linezolid conjugates hijack the ABC (CebE/MsiK) system in *E. coli* and are hydrolyzed by β-glucosidases BglA/BglB; the MIC drops 32-fold, which is confirmed in ΔbglA mutants [97].

Flocculosin, a glycolipid composed of cellobiose linked to a lipid core, exemplifies a dual-action THS: cellobiose motifs hijack ABC transporters for intracellular delivery, while the lipid moiety destabilizes the outer membrane, resulting in 8- to 32-fold reductions in MIC against Gram-negative pathogens [103].

Several approaches have exploited cellobiose and its polymeric form, cellulose, as delivery platforms (Table 1). Upon contact of the functionalized cellulose with microorganisms, due to the specific interaction of cellobiose molecules, the immobilized active compounds occur in close proximity to the cells. The Cel-TDTMABr material (with tetradecyl-trimethylammonium bromide) showed the best antimicrobial activity. Even at a minimal degree of functionalization (ratio of 1:0.012), it provided 100% inhibition of growth for *S. aureus*, *Escherichia coli*, and *Candida albicans* [105]. Functionalized cellulose fibers grafted with β-cyclodextrin and loaded with ciprofloxacin provided sustained antibacterial activity against *E. coli* for up to 15 days (compared to 3 days for the control), and against *S. aureus* up to 11 days (compared to 4 days) [30]. With a modified fiber content of 50% or higher, the growth of both bacteria was completely suppressed (100% inhibition). In this case the antibiotic is released in a controlled manner in close proximity to the bacterial cells, providing a long-lasting antimicrobial effect [30]. Nanocrystalline cellulose conjugates with photosensitizers (e.g., protoporphyrin IX) achieved complete bacterial inactivation upon light activation by concentrating the toxic payload in close proximity to adherent bacteria [31]. The material achieved complete inactivation (99.999+%, a 5-log reduction) of both Gram-positive (*S. aureus*) and Gram-negative (*E. coli*) bacteria in just 20 min of irradiation [31].

Additionally, nanocomposites of cellulose with selenium nanoparticles demonstrated broad-spectrum antimicrobial activity, with the cellulose matrix acting as an inert carrier that delivers the active cargo to the bacterial surface [100]. The composite showed the best antimicrobial activity among all tested samples, surpassing the individual components. For example, the zone of growth inhibition for *Candida albicans* reached 18 mm, and the MIC for this fungus was only 15.6 μg/mL. The composite also effectively inhibited *E. coli*, *P. aeruginosa*, *S. aureus*, and *B. subtilis*, whereas *Aspergillus fumigatus* proved resistant [104].

Application of nanocrystalline cellulose (NCC) as an inert and safe carrier for TDD, including for combating bacteria through various chemical, physical, and enzymatic modifications of NCC, has been recently reviewed by Babaei-Ghazvini et al. [106]. Its unique properties, such as its enormous specific surface area, abundance of surface hydroxyl groups, and nanoscale structure, make it an ideal platform for “loading” with antibacterial “payloads”: traditional antibiotics (ampicillin, tetracycline), antiseptics (triclosan), and natural antimicrobial compounds (curcumin). This approach allows overcoming problems such as the low solubility of hydrophobic antibiotics and their rapid degradation. Thanks to the possibility of fine-tuning the surface, it can carry a diverse range of antibacterial cargoes, ensure their targeted delivery, and enable controlled release directly to the infection site, opening new prospects in the fight against bacterial diseases, including resistant forms [106]. Collectively, these studies demonstrate that cellobiose and cellulose form a unified hierarchical platform for the THS, enabling both active intracellular delivery via ABC transporters and localized extracellular action through nanostructured carriers.

Maltose

Maltose, a disaccharide of two glucose units, is transported by bacteria via high-affinity ABC transporters (MalE–MalF–MalG–MalK) that are absent in mammalian cells [107]. In Gram-negative bacteria, the LamB porin facilitates outer membrane passage, while the periplasmic maltose-binding protein (MalE) captures maltose with exceptional affinity and delivers it to the inner membrane transporter [108]. Gram-positive bacteria utilize a dedicated phosphotransferase system (PTS) for maltose uptake [109]. Human cells lack these systems; maltose is instead hydrolyzed to glucose by intestinal maltase, with glucose subsequently imported via GLUT transporters [110,111].

The versatility of maltose in THSs extends beyond simple antibiotic conjugation. Maltose-based conjugates have been employed as a “bait” for targeted delivery, as “keys” for inducing antimicrobial production, as “alarm clocks” for reactivating dormant persisters, and as “beacons” for diagnostic imaging (Table 3).

Direct conjugation of antibiotics to maltose or maltodextrins enhances bacterial uptake via hijacking the maltose transport system. A ciprofloxacin–maltose conjugate inhibited *E. coli* growth 5- to 25-fold more effectively than free ciprofloxacin [112]. Notably, a maltotriose–perylene conjugate (Cpd-1) was shown to cross both membranes of *E. coli* via LamB and the ABC transporter, and even induced expression of its own uptake machinery [113]. Trimethoprim conjugated to thiolmaltose via a disulfide linker (TM-TMP) achieved >250-fold increased solubility, resisted hydrolysis by host maltase, and released the active drug upon intracellular glutathione cleavage [114]. This approach not only maintains potent antibacterial efficacy—achieving sterile urine in infected mice comparable to the parent drug—but also offers significant advantages in biocompatibility, reduced systemic toxicity, and the ability to overcome permeability-based resistance mechanisms by actively transporting the otherwise poorly permeable antibiotic across the bacterial cell envelope [114].

Maltose has been incorporated into multifunctional prodrugs such as MMCC (mannose–maltose–colistin), where it serves as a bacterial “navigator” for colistin delivery, reducing nephrotoxicity while maintaining efficacy against intracellular *Klebsiella pneumoniae* [115].

Maltose was used to deliver the thiosemicarbazone pharmacophore directly to bacteria [117]. The conjugates exhibited broad-spectrum activity against both Gram-positive (including MRSA) and Gram-negative pathogens, with MICs as low as 0.78 μg/mL—comparable to ciprofloxacin and vancomycin. Importantly, all highly active compounds showed low cytotoxicity against human fibroblasts, demonstrating that maltose enables selective delivery and reduced toxicity [117].

Apart from the direct application of maltose in the form of conjugates for THSs, it has also been used for bacterial cell sensitization to antibacterials. In particular, in an innovative approach, maltodextrin was incorporated into ROS-responsive nanoparticles (MDNPs) and used to awaken dormant *S. aureus* within macrophages, rendering them susceptible to rifampicin [116]. This example illustrates how nanocarrier design can address the challenge of bacterial persisters by combining a metabolic trigger (maltodextrin) with stimuli-responsive drug release.

Maltose has also been employed as an inexpensive trigger for recombinant antimicrobial peptide production in *Bacillus subtilis* [121]. A strain of *Bacillus subtilis* carrying the gene for the antimicrobial peptide T9W (effective against the dangerous pathogen *P. aeruginosa*), which is controlled by the Pglv promoter, activated by maltose was engineered. When maltose was added to the growth medium, it penetrated the *B. subtilis* cells and switched on the promoter, forcing the producer bacterium to synthesize and secrete the target T9W peptide [121].

The maltose-inducible system described above represents an emerging type of THS-bioengineered bacteria [121]. More broadly, probiotic bacteria can be engineered to sense pathogen-specific signals and respond by producing antimicrobials directly at infection sites. While still in early stages, this approach could enable prophylactic or therapeutic intervention against pathogens like *Clostridioides difficile* or enterotoxigenic *E. coli* within the gut, with maltose or other bacteria-specific sugars serving as inducers. Several recent studies have successfully implemented this concept. For example, Koh et al. engineered *E. coli* Nissle 1917 (EcN) with a genetic circuit that restores intestinal bile salt metabolism in response to antibiotic-induced microbiome dysbiosis, significantly reducing *C. difficile* infection in a mouse model (100% survival rate) [122]. Similarly, Li et al. developed an EcN-based “AND logic gate” system that specifically detects and eradicates dual infections of *Pseudomonas aeruginosa* and *Yersinia enterocolitica* upon sensing their respective quorum-sensing molecules, demonstrating both preventive and therapeutic efficacy in vivo [123]. A recent review by Carolak et al. (2025) systematically discusses the broader landscape of genetically engineered probiotics for combating intestinal pathogens, highlighting systems that utilize engineered sensing mechanisms for targeted antimicrobial delivery [124]. These proof-of-concept studies collectively validate the feasibility of using engineered probiotics as living “Trojan Horses” for targeted antimicrobial intervention in the gut, with bacteria-specific sugars and quorum-sensing molecules serving as versatile inducers. Thus, maltose represents a remarkably versatile molecular tool for the THS due to its ability to function as a targeting ligand, a prodrug scaffold, a metabolic trigger, and a diagnostic beacon stem [118,119,120], arising from the unique combination of high-affinity bacterial transport systems and the absence of analogous pathways in mammalian cells. This multifaceted utility positions maltose as a promising platform for developing next-generation antibacterial agents capable of overcoming resistance, targeting intracellular reservoirs, and enabling theranostic applications.

Galactose

Bacteria utilize multiple systems for galactose transport, including ABC transporters (Mgl system), the phosphotransferase system (PTS), and the lactose permease LacY [99,125,126,127,128]. In humans, galactose absorption in the small intestine occurs primarily via the sodium–glucose cotransporter SGLT1 and the facilitative transporter GLUT2 [129]. Although galactose itself is not uniquely bacterial, the specificity of its molecular recognition on pathogen surfaces—particularly by bacterial lectins and adhesins—enables selective targeting. Thus, unlike strategies that rely on active import into the bacterial cytoplasm, galactose-based THSs (Table 4) primarily exploit high-affinity binding to surface-exposed bacterial proteins. This binding can block virulence mechanisms (e.g., adhesion and biofilm formation) while delivering therapeutic payloads directly to the pathogen surface [130].

The most developed galactose-based strategy targets *P. aeruginosa* lectins, particularly LecA, which specifically binds D-galactose. Galactose clusters (galactoclusters) conjugated with siderophore groups enhance both binding to LecA and active transport via the bacterial iron uptake system, improving anti-pseudomonal activity compared to galactose ligands alone [131]. Lactose (a glucose–galactose disaccharide) has been employed in various delivery systems where galactose residues serve as the targeting moiety. Thus, microspheres containing gentamicin, with a matrix of lactose-modified albumin, bind to K88 fimbriae of enterotoxigenic *E. coli* via exposed galactose residues, delivering antibiotics directly to the bacteria [132]. Similarly, lactose-coated nanoparticles loaded with ciprofloxacin demonstrated effective antibacterial activity against *S. aureus* and *P. aeruginosa*, with the lactose matrix attracting bacteria while ensuring rapid drug delivery [134]. Another hypoxia-responsive nanocarrier modified with lactose (LacAC4A) and loaded with ciprofloxacin leverages galactose-mediated targeting of *P. aeruginosa* LecA. Upon bacterial uptake and exposure to hypoxic conditions characteristic of infected wounds, the carrier disassembles, releasing the antibiotic directly inside target cells. This system demonstrated enhanced efficacy against multidrug-resistant *P. aeruginosa* and accelerated healing of infected diabetic wounds in vivo [11].

Application of galactose conjugates as photosensitizer was explored by synthesis of the molecule in which three galactose residues used as targeting “bait” were combined with BODIPY (boron dipyrromethene/4,4-difluoro-4-bora-3a,4a-diaza-s-indacene) photosensitizers that generate reactive oxygen species upon light activation. These conjugates exhibited significant light-induced bactericidal activity against both *P. aeruginosa* and *S. aureus*, demonstrating the potential of galactose for simultaneous imaging and photodynamic therapy [133].

In summary, galactose represents an effective and versatile tool for implementing the THS against bacteria, functioning primarily through high-affinity binding to bacterial surface lectins rather than active import. Its effectiveness stems from the presence of specialized transport and binding systems (ABC transporters, PTS, LacY, lectins) in pathogens, which can be “deceived” using galactose conjugates. Modern applications range from targeted antibiotic delivery and anti-adhesive therapies to theranostic systems combining imaging with photodynamic therapy. Advances in enzymatic synthesis of complex galactose structures [135] further enhance the accessibility and scalability of this strategy for clinical applications.

Raffinose

Raffinose is a non-reducing trisaccharide (galactose–glucose–fructose) that is transported by bacteria via specialized ABC transporters, with expression regulated by catabolite repression [101,102,136]. Due to the presence of a raffinose-binding protein, the transport system is specifically tuned to this trisaccharide. This allows bacteria to selectively absorb raffinose even in the presence of other substances. In Gram-negative bacteria, porins such as RafY facilitate raffinose diffusion across the outer membrane at low substrate concentrations [101].

Humans lack specific transporters for raffinose: it reaches the large intestine intact, where it is fermented by gut microbiota [137]. This differential handling makes raffinose a potentially selective vector for bacterial targeting, though its application in THSs remains underexplored (Table 5).

An innovative application of raffinose in the context of the THS (whilst indirect) involves its use as a prebiotic to selectively stimulate probiotic bacteria that produce antimicrobial agents. In the study by Zhou et al. (2026) [138], raffinose was added to fermented milk to promote the growth of *Limosilactobacillus reuteri* HLRE05, enhancing the production of reuterin—a potent antimicrobial compound—while mitigating its toxic effects on the starter culture. The resulting system provided robust protection against *S. aureus* biofilms and virulence throughout storage, demonstrating a novel approach where raffinose acts not as a passive courier but as an active component that cultivates “saboteur” bacteria and enhances their weaponry directly within the food matrix.

Following this study, more direct bioengineered approaches are also emerging, where commensal bacteria are genetically modified to express and secrete antimicrobial peptides in response to specific triggers [139]. The raffinose–ABC transporter system, which is highly selective for this trisaccharide, could potentially be repurposed as an inducible promoter system in engineered probiotic strains, enabling precise control of antimicrobial production.

In general, raffinose represents an underexplored but promising vector for THSs. While direct conjugation of raffinose to antibiotics or therapeutic payloads has not yet been realized, its selective transport by bacteria and absence of mammalian uptake systems make it an attractive candidate. The demonstrated success of raffinose as a prebiotic activator of antimicrobial production highlights its potential utility in infection control. Future research should focus on developing raffinose–drug conjugates that leverage bacterial ABC transporters for targeted delivery, building upon the extensive knowledge gained from maltose and other sugar-based systems.

Lactose

Lactose (milk sugar) is a disaccharide composed of β-D-galactose and α-D-glucose linked by a β(1 → 4) glycosidic bond. Bacteria utilize specialized transport systems, primarily the LacY lactose permease, which functions as a proton symporter [140,141]. Production of LacY protein is tightly regulated by the *lac* operon and induced only in the absence of glucose [142]. Humans digest lactose in the small intestine via the enzyme lactase; individuals with lactase deficiency cannot absorb intact lactose, which instead reaches the colon for bacterial fermentation.

Lactose serves as a highly effective and multifunctional tool in THSs, performing three key roles: (1) as a “bait” molecule for active targeting of pathogenic bacteria through natural receptors and transport systems; (2) as a biocompatible protective matrix or coating that masks antibacterial cargo and optimizes delivery to infection sites; and (3) as a component of “smart” nanosystems that enable controlled drug release in response to bacterial microenvironment signals (Table 6).

Thus, two lactose esters with unsaturated fatty acids (palmitoleate and nerovonate) embody the THS: lactose acts as a hydrophilic carrier ensuring biocompatibility, while lipophilic fatty acids serve as membrane-disrupting antimicrobial payloads. Both esters exhibited pronounced activity against eight pathogenic microorganisms, including *E. coli*, *S. aureus*, and *P. aeruginosa*, with MIC values of 64–128 μg/mL—significantly more effective than traditional preservatives [144].

Electrosprayed amorphous lactose microparticles encapsulating ciprofloxacin retained full antibacterial activity against *S. aureus* and *P. aeruginosa*, with “empty” lactose particles even stimulating bacterial growth—confirming their role as an attractive nutrient “bait” that ensures rapid drug delivery to the infection site [134]. Another example of lactose usage as a “bait” is the development of lactose-modified azocalixarene nanoparticles (LacAC4A) loaded with ciprofloxacin, enabling active targeting and internalization in *P. aeruginosa*. Under hypoxic conditions characteristic of infected diabetic ulcers, bacterial azoreductases reduce the azo linker, triggering nanoparticle disassembly and intracellular antibiotic release. This system significantly enhanced efficacy against multidrug-resistant *P. aeruginosa*, reduced inflammation, and accelerated wound healing in vivo [11]. The LacAC4A system exemplifies the broader class of stimuli-responsive nanocarriers, where the carrier is designed to release its payload only upon encountering specific bacterial enzymes (azoreductases) or microenvironmental signals (hypoxia). Similar approaches using pH-sensitive or protease-sensitive linkers are being developed for other pathogens and infection sites.

Lactose is widely employed in inhalable formulations due to its ability to form porous microparticles with optimal aerodynamic size for deep lung delivery. Lactose-coated PLGA nanoparticles loaded with *N*-acetylcysteine demonstrated a four-fold increase in antimycobacterial activity compared to the free drug, with lactose serving both as a cryoprotectant and as a mask that evades pulmonary clearance mechanisms [34]. Similarly, spray-dried lactose–leucine microparticles encapsulating anti-tuberculosis nanocarriers enabled effective deposition in the lower respiratory tract, where nanoparticles are subsequently taken up by macrophages—the intracellular niche of *M. tuberculosis* [143].

Thus, lactose represents a highly versatile and effective platform for the THS. Its ability to function as a targeting ligand (via LecA and other lactose-recognizing receptors), a biocompatible carrier matrix, and a component of stimuli-responsive nanosystems enables diverse applications ranging from pulmonary delivery to wound healing. The demonstrated efficacy of lactose-based systems against multidrug-resistant pathogens, coupled with their favorable safety profile, positions lactose as a valuable tool for developing next-generation antibacterial therapies.

Trehalose

Trehalose is a disaccharide of two glucose units linked by an α,α-1,1-glycosidic bond. It is transported by many Gram-positive and Gram-negative bacteria via PTS or ABC transporters. In mycobacteria, trehalose plays a critical structural role as a component of the cell wall, while in other bacteria it serves primarily as a nutritional substrate [145]. Critically, trehalose is absent in mammals, providing a fundamental basis for selective bacterial targeting. However, it should be noted that exogenous trehalose can induce hepatic autophagy via the SLC2A8 (GLUT8) transporter, which may impact in vivo specificity [146]. Since trehalose is absent in mammals, the lack of host production provides a fundamental basis for the selective uptake of trehalose-linked compounds by bacterial cells inside a microorganism in accordance with the THS (Table 7). This principle was suggested and experimentally verified using macrophages infected with *M. tuberculosis* (*Mtb*) [147]. The ability of trehalose conjugates to access the mycobacterial periplasm is a significant advantage. This opens the door for designing small molecules that can specifically target functional domains of transmembrane proteins and inhibit their activity. This design approach is not exclusive to mycobacteria and holds potential for application across various other pathogens [145].

Structural modifications of trehalose, including the creation of conjugates with various compounds, have been achieved either purely through chemical methods or by utilizing natural enzymatic systems involved in trehalose transformations [160].

It was discovered that structurally modified trehalose unconjugated analogs inhibit growth and biofilm formation in *Mycobacterium smegmatis* in the micromolar/millimolar range. The essentiality of the trehalose-specific ABC transporter for this antimicrobial and anti-biofilm activity proves that it is the analogs transported intracellularly that cause the inhibition of cell replication, evidently by inhibiting trehalose metabolism [161].

Among the first trehalose conjugates developed were fluorescent compounds used for the detection of mycobacteria [147]. Subsequent development in this area occurred because the method enables observation in the pathogen’s natural environment, thereby providing innovative prospects for the targeted identification and management of mycobacterial pathogens within intricate biological frameworks [151,153,154]. Specifically, DMN-Tre (a conjugate pairing the fluorogenic dye 4-N,N-Dimethylamino-1,8-naphthalimide with trehalose) selectively targets the mycobacterial cell wall. This selectivity establishes DMN-Tre as a valuable probe for investigating *M. tuberculosis* physiology, applicable both in laboratory culture and within host cells [148,149]. The use of DMN-Tre enabled the rapid detection of *Mtb* in macrophages and patient sputum samples [150,151,152].

Development efforts resulted in a trehalose probe incorporating a 3-hydroxychromone (3HC-3) dye. This construct exhibits superior signal strength, boasting a 10-fold increase in fluorescence intensity relative to the DMN-Tre conjugate [150]. To achieve specific labeling of single, viable *Bacille Calmette-Guérin* (BCG) cells residing within macrophages, a fluorescent probe, cephalosphorinase-dependent green trehalose (CDG-Tre), was engineered. This probe exhibited excellent selectivity for mycobacteria, distinguishing them effectively from other species within the *Corynebacterineae* suborder [151]. The utility of trehalose derivatized carbazole (Tre-Cz) for selectively identifying mycobacteria was demonstrated. The presence of trehalose within this conjugate was key to its ability to detect mycobacteria across complex matrices, including mixed cultures and patient sputum [155].

The potential for transporting low-molecular-weight compounds conjugated to trehalose could potentially be utilized to enhance the delivery of established antibiotics into bacterial cells (especially those with impaired intracellular uptake). However, this concept has only recently been realized in recent work published in 2025. Trehalose conjugates of polyketide synthase 13 (Pks13) inhibitors demonstrated that the attachment of trehalose served to significantly enhance either the antimycobacterial potency or improve selectivity (by reducing toxicity) of the Pks13 inhibitors against *M. smegmatis* and *M. tuberculosis* [156].

At the same time, there are several publications reporting the use of trehalose conjugated with photosensitizers to sensitize bacterial cells for photodynamic inactivation. For example, a trehalose conjugate with BODIPY acting as the photosensitizer (PS) was found to have a minimum inhibitory concentration (MIC) for its photoactivity against *M. smegmatis* and *M. abscessus* in the range of 0.5–33 µM [157]. With the trehalose–porphyrin conjugate, only a single concentration (10mM) was tested, yet this concentration successfully yielded a noticeable inhibition of *M. smegmatis* [158]. In our own experiments, we assessed the antibacterial activity of trehalose conjugates with tricarbocyanine (TCC2Tre) following photodynamic inactivation (PDI) against mycobacteria. We determined that 20 µM of TCC2Tre was sufficient to achieve 99.9% photoinactivation of both *M. smegmatis* and *M. tuberculosis*. Furthermore, a 40 µM concentration of TCC2Tre produced a significant killing effect on *M. tuberculosis*. Crucially, the trehalose-free photosensitizer exhibited substantially lower activity compared to its trehalose-conjugated counterpart when subjected to photoactivation [159].

In summary, trehalose represents a highly selective vector for targeting mycobacteria, leveraging the absence of this sugar in mammals and its essential role in mycobacterial cell wall biology. Trehalose-based probes have enabled sensitive detection of *M. tuberculosis* in clinical samples, while trehalose–photosensitizer conjugates offer a promising approach for photodynamic therapy of mycobacterial infections. Trehalose conjugates with antibacterials are of potential effectiveness; however this possibility has not been substantially explored so far. Future research should focus on overcoming trehalase-mediated degradation in vivo through the development of non-digestible trehalose analogs and expanding the application of this strategy to other bacterial pathogens that utilize trehalose as a nutrient or structural component.

Mannose

Mannose is a hexose monosaccharide that is transported by bacteria such as *E. coli* and streptococci via the PTS [162]. In mammals, mannose plays a distinct role: rather than serving as a carbon source, it is recognized by mannose receptors (MR, CD206) expressed on the surface of macrophages and other immune cells. These C-type lectin receptors are key components of innate immunity, binding mannose residues on bacterial, fungal, and other pathogen surfaces [163]. This dual recognition—bacterial uptake via PTS and mammalian binding via mannose receptors—enables mannose to function in the THS through two complementary mechanisms: (1) direct targeting of bacterial adhesins to block infection and deliver payloads, and (2) targeting of host macrophages to deliver therapeutics into the intracellular niches where pathogens such as *M. tuberculosis* reside (Table 8). Thus, nanoparticles or proteins coated with mannose are selectively absorbed by macrophages, which allows for the treatment of intracellular infections like tuberculosis [164], minimizing side effects for the rest of the body. Mannose-coated PLGA–PEG nanoparticles loaded with rifapentine achieved a four-fold reduction in effective drug dose compared to the free drug and provided sustained release for up to 60 h, enhancing intracellular killing of *M. tuberculosis* [165]. Mannose-modified lipid nanoparticles have similarly been used for targeted rifampicin delivery in tuberculosis treatment [166].

In another approach, mannose-functionalized nanoparticles with photothermal properties (MP-MENP) have been developed to target circulating monocytes and infiltrating macrophages at infection sites. Upon laser irradiation, these nanoparticles generate heat, destroying both extracellular and intracellular *S. aureus* (including MRSA) while triggering an immune response [167].

Mannose binds with high affinity to bacterial adhesins such as FimH in uropathogenic *E. coli*, blocking bacterial attachment to host cells and disrupting biofilms [168]. This property has been exploited in diagnostic applications: an optical sensor based on mannose-functionalized emulsion droplets detects *Salmonella enterica* by competitive displacement of mannan from the droplet surface upon bacterial binding, enabling rapid detection (1 h) with sensitivity down to 100 cells/mL [169].

Thus, mannose is a versatile tool for the THS, functioning through two distinct but complementary mechanisms exploring mannose receptors in macrophages and binding with bacterial adhesins. This dual functionality, combined with its biocompatibility and ease of chemical modification, positions mannose as an extremely promising platform for developing targeted antimicrobial therapies and diagnostic tools capable of overcoming multidrug resistance and reducing systemic toxicity. Future directions may include the development of dual-targeting constructs that simultaneously engage both bacterial and host receptors for enhanced selectivity and efficacy.

Sugar acids and their phosphorylated forms, glucans

Sugar acids, such as gluconate and glucuronate, are imported into bacterial cells via specific active transport systems (e.g., the Gnt family of H^+^ symporters) and are subsequently phosphorylated to enter central carbon metabolism via the Entner–Doudoroff pathway or the pentose phosphate pathway [170,171,172,173]. These pathways are highly conserved in many bacteria, including *E. coli*, *Pseudomonas* species, and mycobacteria [174,175,176]. In contrast, mammals lack dedicated gluconate transporters and gluconokinases, and do not utilize the Entner–Doudoroff pathway [177]. This differential metabolism makes sugar acids attractive vectors for bacterial targeting.

D-glucuronic acid is a component of bacterial polysaccharides, utilized by many bacterial species, and most importantly, serves as a substrate for specific bacterial enzymes— β-glucuronidases. Many bacteria, including *E. coli*, *Salmonella*, and *Clostridium*, produce β-glucuronidases—enzymes that hydrolyze the glucuronide bond. This capability allows for the design of prodrugs that are activated only inside or in the immediate vicinity of the bacterial cell (Table 9).

The ERNathG probe (4-hydroxy-1,8-naphthalimide conjugated with glucuronic acid) exemplifies the THS using glucuronic acid as a recognition site for bacterial β-glucuronidase (GUS). Upon bacterial uptake, GUS hydrolyzes the glucuronic acid–fluorophore bond, releasing a brightly fluorescent molecule. The probe selectively stains GUS-positive bacteria (*E. coli*, *S. warneri*) while leaving GUS-negative bacteria (*S. aureus*) undetected, with sensitivity down to 1 CFU/mL (colony-forming unit per milliliter) [178].

Glucuronic acid has been used to functionalize mesoporous silica nanoparticles (GLY-MSN) for enhanced bacterial uptake and antibiotic delivery. These glycosylated nanoparticles demonstrated significantly improved antibacterial efficacy compared to non-targeted formulations [179].

Glucuronic acid is one of the key building blocks of many complex glucans (polysaccharides composed of glucose monomers). The transport of glucans into bacteria occurs predominantly through the PTS for short soluble oligo-/polyglycosides (maltodextrins, β-glucans) but ABC transporters are used for long-chain molecules, involving phosphorylation for accumulation inside the cell [99]. Labeled glucans have been utilized primarily as carriers for targeted delivery to macrophages rather than for direct bacterial uptake (Table 9). Thus, Glucan Lipid Particles (GLPs) encapsulating anti-tuberculosis drugs (clofazimine, isoniazid, linezolid) enable targeted delivery to the lungs and recognition by macrophages, concentrating therapeutics at the site of mycobacterial infection [180].

Fluorescently labeled polysaccharides (FLAPSs) have been developed to visualize bacterial carbohydrate uptake in real time, with bacteria importing these probes via specialized transport systems (e.g., SusCD-like complexes in Bacteroidetes) [181].

Summarizing, while gluconate has not yet been experimentally exploited for antibiotic delivery, its high-affinity active transport systems in bacteria make it a theoretically promising vector. The existence of dedicated, regulated permeases (e.g., GntT, GntU) that actively import gluconate against concentration gradients provides an ideal “gateway” for Trojan Horse conjugates. Future research should focus on developing gluconate–antibiotic conjugates that leverage these transport systems for targeted delivery. Glucuronic acid has proven effective as a targeting moiety for diagnostic probes and antibiotic delivery, leveraging bacterial β-glucuronidase activity for selective activation. Glucans serve primarily as carriers for macrophage-targeted delivery, concentrating therapeutics at intracellular infection sites. The growing toolkit of sugar acid-based probes and delivery systems underscores the potential of these metabolites for developing selective antibacterial diagnostics and therapeutics.

β-Glycosides (specific to bacteria)

β-Glycosides are compounds in which a sugar moiety (glycone) is linked to a non-proteinaceous aglycone via a β-glycosidic bond [182]. Common examples of specific for bacteria glycosides include salicin (a salicyl alcohol glucoside), arbutin (a hydroquinone glucoside), and cellobiose (β(1 → 6)-glucose dimer) [183,184,185]. Bacteria possess specialized systems for β-glycoside utilization, primary the PTS [186]. The bacterial PTS for β-glycosides involves a phosphorylation cascade that couples transport with substrate modification, ensuring efficient intracellular accumulation [186]. This system is particularly attractive for the THS because it is absent in eukaryotes [100] and is essential for bacterial utilization of these compounds. β-Glycosides have been employed as carriers for antibacterial prodrugs that are activated by bacterial enzymes (Table 10). The β-glucosidic linkage serves as a cleavable bond that is hydrolyzed by bacterial β-glucosidases, releasing the active payload specifically within bacterial cells. This approach minimizes off-target effects and enhances selectivity.

The expression of bacterial β-glycoside transport and metabolism is tightly regulated, often induced by the presence of the substrate and repressed by preferred carbon sources such as glucose [186]. This regulation must be considered when designing β-glycoside-based conjugates to ensure consistent uptake under infection-relevant conditions. Additionally, while mammals lack the bacterial PTS, they do express β-glucosidases (e.g., GBA3) that can hydrolyze β-glycosidic linkages, potentially leading to premature drug activation [188]. Conjugate design should therefore incorporate strategies to minimize susceptibility to host enzymes. As an example of usage of glycosides for the THS, ten glycoside derivatives with the antibacterial compound L-R-aminoethylphosphonic acid (L-AEP), an inhibitor of L-alanine racemase, were synthesized and selectively taken up by bacterial cells through carbohydrate uptake mechanisms [187]. After uptake, the prodrugs were hydrolyzed by bacterial glycosidases, releasing active L-AEP. β-Glycosides, containing L-AEP linked to glucose or galactose via a carbamate bond, demonstrated growth inhibition of a broad spectrum of Gram-negative bacteria (MIC < 0.75 mg/mL), with inhibition correlating to the hydrolysis of the corresponding chromogenic glycosides [187].

A key feature that makes bacterial β-glycosides (such as salicin, arbutin, and cellobiose) attractive vectors for the THS is their selective uptake by bacterial cells. Mammalian cells are incapable of actively transporting these compounds in their intact form. This limitation stems from a fundamental difference in carbohydrate metabolism: mammals lack the PTS [85,87]—a unique bacterial machinery that couples β-glycoside transport with phosphorylation [186]. Without the PTS, these molecules cannot be actively imported across the host cell plasma membrane. In mammals, β-glycosides are typically hydrolyzed by enzymes of the gut microbiota rather than being transported into host tissues. Although mammals express β-glucosidases (e.g., GBA3) capable of hydrolyzing β-glycosidic linkages, these enzymes are localized intracellularly (in lysosomes and the endoplasmic reticulum) and do not facilitate active import of intact molecules [188]. Moreover, the substrate specificity of mammalian enzymes differs substantially from bacterial PTS-dependent systems [100]. Consequently, conjugates based on such glycosides exhibit high selectivity for bacteria, minimizing off-target effects on host cells and enabling the development of safe, targeted antibacterial therapies. Notable exceptions, such as the entry mechanism of cholera toxin that exploits alternative endocytic pathways, only confirm the general rule and do not overturn the absence of direct active import of bacterial β-glycosides by mammalian cells.

β-Glycosides represent an almost underexplored but promising class of vectors for the THS. Their selective recognition by bacterial PTSs and susceptibility to bacterial β-glucosidases offer multiple opportunities for targeted drug delivery. Future research should focus on: (1) expanding the repertoire of β-glycoside scaffolds; (2) developing conjugates with cleavable linkers that are stable in circulation but efficiently processed by bacterial enzymes; and (3) evaluating the impact of host β-glucosidases on conjugate stability and selectivity.

Non-metabolizable sugar analogs

Non-metabolizable sugar analogs are synthetic derivatives of natural sugars that are transported into bacteria via specific uptake systems but cannot be further metabolized by bacterial enzymes. This property offers several advantages for the THS: (1) they accumulate inside bacterial cells, enhancing signal or payload concentration; (2) they are resistant to bacterial metabolic degradation; and (3) they are often poorly recognized by mammalian transporters, improving selectivity. Classic non-metabolizable glucose analogs such as 2-deoxyglucose (2-DG) and α-methylglucoside are transported by bacterial PTSs or ABC systems but cannot be phosphorylated or further processed, leading to intracellular accumulation [189]. Similarly, methyl-β-D-thiogalactopyranoside (TMG) is a thio-analog of galactoside that is transported but not metabolized, making it a valuable tool for studying sugar transport [190].

Rare sugar such as D-allulose is metabolized only by a limited number of bacteria (via the enzyme AlsE) but not by humans, enabling species-specific targeting [191]. D-lyxose is another example: while not normally assimilated by *E. coli*, it can be transported via the D-xylose system, allowing selective delivery to strains that express the appropriate transporters [192]. Despite potential applicability of the abovementioned natural compounds for the THS, experimental studies in this regard are absent so far. At the same time, several chemically modified sugars revealed their ability to transport into bacterial cell (Table 11). In particular, azide-modified sugars (AMSs) such as GalNAz, ManNAz, and GlcNAz, which are synthetic analogs of natural sugars, were able to be incorporated into bacterial cell wall polysaccharides via endogenous metabolic pathways. The azide group serves as a bioorthogonal “handle” for subsequent click chemistry with fluorescent labels, enabling selective visualization of bacteria in complex environments such as the gut microbiota [193]. This approach allows real-time tracking of bacteria without disrupting viability.

Fluorine-18-labeled disaccharides derived from [^18^F]FDG via chemoenzymatic synthesis—such as ^18^F-FDM (α-1,4-linked maltose) and ^18^F-FSK (α-1,3-linked sakebiose)—serve as non-metabolizable sugar analogs for positron emission tomography (PET) imaging of bacterial infections. These tracers are selectively taken up by bacteria via maltodextrin transporters, accumulate intracellularly due to their inability to be fully metabolized, and enable specific visualization of infections in vivo, distinguishing them from sterile inflammation [194].

Non-metabolizable sugar analogs potentially offer several key advantages for the THS. They exploit bacterial transport systems with high specificity and accumulate intracellularly due to metabolic trapping, thereby enhancing payload concentration.

In order to confirm applicability of non-metabolizable sugar analogs for the THS, future research should focus on developing novel analogs with improved selectivity and metabolic stability, conjugating them with therapeutic payloads for targeted antibiotic delivery, and validating these platforms in clinically relevant infection models to support translation.

##### Components of Bacterial Cell Walls

Peptidoglycan (murein) (PG) is the primary structural component of the bacterial cell wall and is entirely absent in human cells. This makes the transport of its components (e.g., muramic acid, D-amino acids) potentially more efficient in bacteria. Most bacteria actively recycle their peptidoglycan [195]. During cell growth and division, peptidoglycan fragments are released into the environment and subsequently transported back into the cytoplasm for reuse [196]. Bacteria utilize specialized transporter proteins, such as AmpG, OppBCDF-MppA, and YejBEF-YepA, for the import of PG fragments [195]. There are transporters for specific components, such as MurP for the transport of *N*-acetylmuramic acid (MurNAc) [197]. Some bacteria, such as the oral pathogen *Tannerella forsythia*, have lost the genes for MurNAc synthesis and are entirely dependent on importing it from the outside (e.g., from PG fragments of other bacteria) [198], making this transport pathway a vulnerable target.

*N*-acetylglucosamine (GlcNAc) and Glucosamine (GlcN)

*N*-acetylglucosamine (GlcNAc) is a structural component of bacterial cell wall peptidoglycans, where it alternates with *N*-acetylmuramic acid (MurNAc) to form glycan chains [199,200,201].

Free GlcNAc is released during cell wall turnover and is taken up by bacteria via specific transport systems. In *E. coli*, GlcNAc import is mediated by the PTS transporter NagE, which phosphorylates the GlcNAc during transport [202]. Other bacteria, such as actinomycetes, utilize ABC transporters for GlcNAc uptake [203]. Importantly, the metabolic pathway for GlcNAc differs between prokaryotes and eukaryotes: bacteria synthesize GlcNAc-1-P via a unique route, while eukaryotes produce GlcNAc-6-P [200,204]. This distinction, combined with the absence of free GlcNAc in mammalian cells under normal conditions, provides a basis for selective bacterial targeting.

Glucosamine (GlcN), the deacetylated form of GlcNAc, is also transported by bacteria. A breakthrough in understanding GlcN transport came with the characterization of the ABC transporter substrate-binding protein Avi_5305 from *Agrobacterium vitis*, which specifically binds D-glucosamine and D-galactosamine via a cation–π interaction with Tyr168 [205].

Several studies have exploited these amino sugars for the THS (Table 12). Conjugates of fluoroquinolone antibiotics (ciprofloxacin, norfloxacin, moxifloxacin) with glucosamine demonstrated enhanced activity against both Gram-positive and Gram-negative bacteria, including fluoroquinolone-resistant *E. coli*, as well as certain fungi [206,207]. Notably, these conjugates exhibited 100-fold lower cytotoxicity than free fluoroquinolones, suggesting that bacterial PTSs mediate selective uptake, while eukaryotic transporters are less efficient for conjugated compounds [206,207]. Glucosamine–fluoroquinolone conjugates also showed improved environmental biodegradability compared to parent antibiotics [208].

Glucosamine–lactate copolymers have been developed as particles for sustained rifampicin release. In these particles, surface-exposed glucosamine has beem hypothesized to enhance binding to target bacteria, as demonstrated for *Mycobacterium smegmatis* experimentally [209]. Additionally, GlcNAc has been incorporated into glycoconjugate vaccines against Group A *Streptococcus* infection, where GlcNAc serves as a key antigenic determinant in the Group A Carbohydrate (GAC) [210,211].

GlcNAc and GlcN offer selective targeting opportunities for the THS due to differences in bacterial versus mammalian metabolism and transport. While both amino sugars can be transported by eukaryotic cells, the lower efficiency of these systems for conjugated compounds enables selective delivery. Future directions include optimizing structure chemistry to further enhance selectivity, increasing the number of antibiotic payloads conjugated to these versatile scaffolds and exploring their performance *in vivo*.

*N*-acetylmuramic acid (MurNAc) and muropeptides

MurNAc, a peptidoglycan component unique to bacteria, is imported via dedicated transporters such as the PTS permease MurP (in *Firmicutes* and *Proteobacteria*) and the MFS transporter AmpG (in Gram-negative bacteria) [212,213]. These systems actively recycle cell wall fragments and provide a selective entry point for drug conjugates.

Several studies have exploited MurNAc transporters for cargo delivery (Table 13). Notably, a GlcNAc–anhydroMurNAc–fluorophore conjugate was shown to enter *E. coli* via AmpG [214], confirming that these transporters tolerate bulky modifications. Similarly, UDP–MurNAc derivatives were used as probes for labeling bacterial peptidoglycan biosynthesis pathways, enabling visualization and study of cell wall dynamics without mammalian interference [215].

Usage muropeptides for a “Trojan Horse” antibiotic strategy presents a significant paradox: the very mechanism that makes them effective vectors for bacterial uptake (their recognition by bacterial importers) also triggers potent immune surveillance in the host, which can limit their application [216]. The primary limitation is that muropeptides are not “stealth” molecules. They are potent pathogen-associated molecular patterns (PAMPs) that are constantly monitored by the host’s innate immune system [216]. Recognition leads to rapid inflammatory response, potentially causing off-target effects or conjugate clearance before reaching bacteria. Muropeptide–antibiotic conjugates could trigger inflammation at the infection site, exacerbating tissue damage. Widespread distribution increases risk of conjugate interaction with host cells, leading to unintended systemic effects. However, a vast array of muropeptide structures exist due to bacterial species variations and modifications (O-acetylation, *N*-deacetylation) [216] and not all muropeptides are pro-inflammatory. Recent research using advanced computational tools (like PGN_MS2) has identified muropeptides from beneficial bacteria (e.g., *Bifidobacterium*) that possess anti-inflammatory activity, suppressing LPS-induced inflammation [216]. If such muropeptides retain the ability to be imported by bacteria, they could serve as “silent” or even “protective” vectors. Medicinal chemistry could be employed to modify the muropeptide structure. The goal would be to retain the molecular features necessary for recognition by bacterial importers while disrupting the epitopes that bind to host NOD receptors. If systemic administration is too risky, the Trojan Horse conjugate could be delivered directly to the site of infection (e.g., topical application for skin infections, or inhaled formulations for lung infections). This could achieve high local concentrations at the bacterial target while minimizing systemic exposure and subsequent immune activation.

Future research should focus on: (1) identifying or engineering muropeptide variants with reduced inflammatory potential while retaining transporter recognition; (2) developing conjugation strategies that mask immunogenic epitopes until delivery; and (3) exploring localized delivery routes (topical, inhaled). The demonstrated ability of AmpG and related transporters to internalize modified substrates [214] provides a strong foundation for developing MurNAc-based antibiotic conjugates with high bacterial specificity.

Arabinose and Arabinooligosaccharides

Arabinose is a pentose sugar that exists in two stereoisomeric forms: D-arabinose and, more common in nature, L-arabinose. For bacteria, arabinose serves not only as a carbon source but also as a critical structural component, especially for mycobacteria, where it participates in the synthesis of arabinogalactan (AG) and lipoarabinomannan—key elements of the cell wall that influence its permeability and antibiotic resistance [217].

The transport of arabinose in bacteria such as *E. coli* is mediated by the products of the *araE*, *araF*, *araG*, and *araH* genes, which are part of the arabinose operon and ensure the uptake of the sugar from the environment [217]. The selectivity of arabinose-based targeting stems from its unique structural roles in bacteria and its absence from mammalian metabolism. D-Arabinofuranose (Araf), in particular, is not found in humans but is present in the cell wall of certain bacteria, providing a basis for highly specific recognition.

Several studies have explored arabinose and arabinooligosaccharides for the THS applications (Table 14). Tetracyclic conjugates linking 1,4-naphthoquinones with thio-derivatives of L-arabinose demonstrated antimicrobial activity comparable to free vancomycin and gentamicin [218]. More importantly, a library of multivalent probes based on D-arabinofuranose revealed that the mode of attachment critically influences targeting specificity: probes with arabinose attached to polymeric microparticles via the C2 position showed the best and most selective binding to *S. aureus*, and were also capable of disrupting biofilm formation [219].

Arabinooligosaccharides, commonly referred to as arabinoxylan-oligosaccharides (AXOSs), are short sugar chain composed of a xylan backbone with arabinose side branches. Certain bacteria possess highly specialized ABC transporters for the uptake of intact AXOSs [220]. These are complex protein assemblies that span the bacterial membrane. They have three main components, but the most important for our purpose is the SBP (substrate-binding protein), which acts as a ”bait” or ”trap” for the particular substrate [220]. SBP in the Gram-positive bacterium *Bifidobacterium animalis* (BlAXBP) captures arabinoxylan-oligosaccharides [221]. BlAXBP possesses exceptionally broad substrate specificity. It can bind oligosaccharides with various arabinose derivatives and even recognize them in two opposite orientations. This is direct proof that the transporter is ‘tolerant’ to modifications of the substrate’s structure, meaning it can, in principle, be used for delivering conjugates where a beneficial molecule is attached to the oligosaccharide. The protein’s binding pocket is spacious enough to accommodate various modifications [221].

After transport, AXOSs are hydrolyzed by enzymes such as arabinosidases, releasing arabinose [220]. The arabinose is then metabolized through various bacterial pathways. Eukaryotic cells lack dedicated transport systems for AXOSs, underscoring the potential of these molecules for the THS. However, this approach has yet to be explored/implemented.

Studies in humans and animal models (rats, pigs) unequivocally show that AXOSs, whose main component is arabinooligosaccharides, are not digested in the small intestine and reach the large intestine intact, where they are fermented by gut bacteria. This process leads to the production of short-chain fatty acids, such as acetate, propionate, and butyrate [222,223].

In general, arabinose and arabinooligosaccharides represent underexplored but promising vectors for the THS. The unique presence of D-arabinofuranose in bacterial cell walls and the existence of highly specific ABC transporters for AXOSs offer opportunities for selective targeting, particularly for gut microbiota and mycobacterial infections. Future research should focus on: (1) developing arabinose–antibiotic conjugates that exploit bacterial arabinose transporters; (2) utilizing the C2-linked arabinose platform for targeted delivery of biofilm-disrupting agents; and (3) exploring AXOS-based conjugates for selective delivery to beneficial or pathogenic gut bacteria. The demonstrated tolerance of arabinose-binding proteins for structural modifications suggests that these systems can accommodate diverse therapeutic payloads.

Arabinogalactan

Arabinogalactan (AG) is a polysaccharide found in higher plants [224], and, importantly, serves as a critical structural component of the mycobacterial cell wall, where it forms the mycolyl–AG–peptidoglycan (mAGP) complex [225,226,227,228]. In mycobacteria, AG links peptidoglycan to the mycolic acid layer, ensuring cell envelope integrity and contributing to the intrinsic resistance of mycobacteria to many antibiotics [227,228]. The unique presence of AG in mycobacteria, combined with its absence in mammals, makes it an attractive target for selective drug delivery.

Two fundamentally different transport mechanisms can be identified, which may be related to its utilization or integration into the bacterial cell wall. In mycobacteria, the ABC transporter Wzm-Wzt is responsible for exporting polysaccharide precursors for cell wall biosynthesis, representing a critical target for disrupting mAGP assembly [229,230]. In environmental bacteria such as *Maribacter* sp., growth on AG induces three TonB-dependent SusC/D-type transporters [231] that capture and import large polysaccharides. These systems, along with associated glycoside hydrolases, enable the utilization of AG as a nutrient source [231].

Beyond serving as a transport substrate, AG has been successfully employed as a biocompatible carrier matrix for antimicrobial delivery. Sulfated AG stabilized silver nanoparticles (Ag(0)) and facilitated their adhesion to bacterial cells, concentrating the antimicrobial cargo directly on the target bacterium. Following delivery, silver ions released from the nanoparticles triggered redox reactions, forming additional nanoparticles on bacterial membranes and enhancing the antimicrobial effect [232]. In a more recent study, water-soluble nanocomposites of AG and elemental iodine were developed using green mechanochemical methods. AG served as a stabilizing matrix, preventing iodine aggregation, ensuring water solubility, and potentially facilitating targeted delivery to microbial cells due to its membranotropic properties. The resulting nanocomposite demonstrated pronounced activity against a broad spectrum of pathogens, including multidrug-resistant *K. pneumoniae* [233].

AG offers a role in the THS as a biocompatible carrier for delivering antimicrobial payloads. Future research should focus on: (1) exploiting the Wzm-Wzt ABC transporter for targeted delivery of antibiotics to mycobacteria; (2) developing AG-based nanocarriers with enhanced selectivity for mycobacterial infections; and (3) optimizing AG conjugation chemistry to improve payload stability and release kinetics. The demonstrated success of AG as a stabilizing matrix for silver and iodine nanoparticles highlights its potential for developing new antimicrobial formulations with improved safety and efficacy profiles.

##### D-Amino Acids

While L-amino acids are the universal building blocks of proteins, D-amino acids play specialized roles in the bacterial world. They function as regulators of biofilm formation, key components of the cell wall (peptidoglycan), and intercellular signaling molecules [234]. Bacteria synthesize over 10 types of D-amino acids, with D-alanine and D-glutamate being the most commonly used for cross-linking in peptidoglycan [234]. *S. aureus* utilizes D-amino acids, particularly D-alanine and D-glutamate, for peptidoglycan biosynthesis. Apart of synthesis, bacteria can import D-amino acids to maintain cell wall integrity and stability [235,236]. Although Gram-negative bacteria have less peptidoglycan in their cell walls than Gram-positive bacteria, D-amino acids are still essential for its synthesis.

The transport of D-amino acids into bacterial cells occurs primarily through two mechanisms. The most universal pathway involves their “masking” within short peptides: broad-spectrum peptide transporters such as Dpp (dipeptide permease) and Opp (oligopeptide permease) import di- and tripeptides containing D-amino acids, which are subsequently cleaved by intracellular peptidases [236,237]. These ABC transporters are notable for their insensitivity to the stereochemistry of individual residues, recognizing the peptide backbone rather than chirality. Additionally, specialized transporters for free D-amino acids also exist such as the CycA system (also known as DagA) in *E. coli*, which functions as an H^+^-symporter capable of transporting D-alanine and its toxic structural homolog D-cycloserine [238].

The unique role of D-amino acids in bacterial physiology has been exploited for Trojan Horse strategies in several innovative ways (Table 15). A metabolic probe combining D-alanine with an aggregation-induced emission (AIE) photosensitizer (TPACN-D-Ala) enabled simultaneous visualization and destruction of bacteria after illumination. The probe is specifically incorporated into bacterial peptidoglycan via the cell wall synthesis machinery, allowing detection and photodynamic killing of biofilms and intracellular *S. aureus* (including MRSA) without affecting host cells [239].

In a proof-of-concept study, unnatural D-amino acid derivatives conjugated to dinitrophenyl (DNP) were used to “mark” bacterial surfaces. Bacterial transpeptidases mistakenly incorporated these conjugates into peptidoglycan, leading to opsonization by anti-DNP antibodies and subsequent immune-mediated killing [240]. This approach demonstrates that D-amino acids can deliver antigenic payloads to the bacterial surface, leveraging the bacterium’s own enzymatic machinery.

**Table 15:** Representative D-amino acid-based Trojan Horse systems.

Platform	Function	Mechanism	Target	Key Outcome
TPACN-D-Ala (D-alanine–AIE photosensitizer)	Theranostic (imaging + photodynamic therapy)	Metabolic incorporation into peptidoglycan	MRSA, biofilms, intracellular *S. aureus*	Simultaneous visualization and killing; spares host cells [239]
Fluorescent D-amino acid derivatives	Metabolic labeling	Incorporation via D,D- and L,D-transpeptidases	Diverse bacteria (*E. coli*, *S. aureus*, etc.)	Visualization of peptidoglycan synthesis; bypasses cytoplasm [241,242]
D-amino acid–DNP conjugates	Immune labeling	Transpeptidase-mediated surface display	*S. aureus*, *E. coli*	Opsonization and immune-mediated killing [240]

In general, fluorescent D-amino acid derivatives have been widely used as metabolic labels for visualizing peptidoglycan synthesis in diverse bacterial species, including *Chlamydia*, *Bacillus subtilis*, *E. coli*, *S. aureus*, and *Lactococcus lactis* [241]. A key mechanistic insight emerged from studies showing that fluorescent D-amino acids are incorporated not via the cytoplasmic pathway but directly in the periplasm by D,D-transpeptidases and L,D-transpeptidases, delivering cargo directly to the site of cell wall assembly [242].

D-amino acids offer unique advantages for the THS: (1) they are incorporated into peptidoglycan via the bacterium’s own cell wall synthesis machinery, enabling covalent surface display; (2) the incorporation occurs directly at the cell wall assembly site, bypassing the cytoplasm; and (3) the peptidoglycan recycling pathway provides continuous opportunity for delivery. However, limitations include the presence of some D-amino acids (e.g., D-serine) in mammals, where they function as neurotransmitters, potentially leading to off-target effects. Additionally, the promiscuity of peptide transporters may reduce selectivity, requiring careful conjugate design. Ongoing research should focus on: (1) creation of D-amino acid conjugates to include a broader range of therapeutic payloads (antibiotics, photosensitizers, immune modulators); (2) improving structure to ensure efficient incorporation while maintaining payload activity; and (3) exploring species-specific differences in D-amino acid metabolism to achieve selective targeting of pathogenic bacteria while sparing commensal microbiota. The demonstrated versatility of D-amino acids as molecular “anchors” for surface display positions them as valuable tools for both therapeutic and diagnostic applications.

Vitamins

Many pathogenic bacteria acquire vitamins from the host environment. To meet this need, they possess highly specific transport proteins that actively import these essential micronutrients. Vitamin conjugates can exploit these natural transport pathways as an approach for THS realization. B-group vitamins (B1, B2, B7, B9, B12) are especially attractive targets due to the high intensity of their bacterial uptake systems.

A number of studies have shown promising prospects for applying this strategy (Table 16).

Vitamin B12 has been extensively explored as a vector for targeted antibiotic delivery. Conjugates of B12 with ampicillin demonstrated 500- and 60-fold higher activity against *E. coli* and *S. typhimurium*, respectively, compared to free ampicillin, and 8-fold higher activity than ciprofloxacin [243]. B12–chloramphenicol conjugates maintained antibacterial efficacy while exhibiting reduced toxicity [243]. B12 has also been used to deliver antisense peptide nucleic acids (PNAs) and oligonucleotides to *E. coli* and *S. typhimurium*, enabling sequence-specific gene silencing [244,245]. Multifunctional systems combining B12 with siderophores and antibiotics (e.g., colistin) have been developed to enable both intravenous and oral delivery [246,253,254].

Riboflavin serves a dual function in the THS: as a photosensitizer for photodynamic therapy and as a photolabile protecting group for controlled antibiotic release. Thus, the VanB2 conjugate (riboflavin–vancomycin) binds to Gram-positive bacteria and, upon blue light exposure, generates reactive oxygen species for rapid photodynamic killing while simultaneously releasing free vancomycin via photoinduced bond cleavage [247]. Riboflavin has also been incorporated into iron oxide nanozymes for wound healing [248] and into microneedles for bacterial keratitis treatment [255].

Vitamin C acts as a multifunctional antimicrobial agent against carbapenem-resistant hypervirulent *Klebsiella pneumoniae* (CR-hvKP). At high doses, it penetrates bacterial cells via PTS [256] and triggers the Fenton reaction (due to high intracellular iron content), generating oxidative stress that damages DNA, lipids, and proteins. At sub-MICs, it inhibits efflux pumps and suppresses biofilm formation and virulence factor expression [249]. Thus, vitamin C exploits the metabolic characteristics of particular bacteria (high iron ion intracellular content in comparison to mammalian cells) producing lethal oxidative damage inside the cell, while simultaneously depriving them of their defense mechanisms [249]. At the same time, this feature is not typical for many bacteria, evidently limiting this approach.

Vitamins (folic acid, B12, biotin, riboflavin, C, D) have been conjugated to metallic nanoparticles (Au, Ag, Fe_3_O_4_, Cu_2_S, CeO_2_) to enhance antimicrobial effects. The vitamin acts as a “bait,” ensuring receptor-mediated uptake via vitamin transporters, while the metallic core delivers the lethal payload—generating reactive oxygen species, releasing toxic ions, or enabling photothermal therapy [252]. Such conjugates have demonstrated potent activity against MRSA and other pathogens while minimizing off-target effects [250,251].

The vitamin–nanoparticle platform is particularly versatile because it combines active targeting (via vitamin transporters) with the multifunctional capabilities of nanomaterials. For example, riboflavin-conjugated gold nanoparticles enable both photothermal therapy (via gold core heating upon laser irradiation) and photodynamic therapy (via ROS generation from riboflavin), creating a synergistic antimicrobial effect [257]. Similarly, folic acid-functionalized silver nanoparticles embedded in metal–organic frameworks (MOFs) provide sustained release and combined antimicrobial activity [258]. These vitamin–nanoparticle conjugates represent a convergence of active targeting and nanocarrier-based drug delivery, offering enhanced efficacy with reduced off-target toxicity.

Vitamin-based THSs offer several key advantages: (1) vitamins are nutrients with high-affinity bacterial transporters; (2) some bacteria are auxotrophic for specific vitamins, making their uptake systems constitutively active; (3) vitamin–antibiotic conjugates can overcome permeability-based resistance; and (4) vitamins themselves can possess intrinsic antimicrobial activity (e.g., riboflavin as a photosensitizer, vitamin C as a pro-oxidant). Limitations include potential competition with host vitamin utilization pathways and the possibility of transporter mutations leading to resistance.

Future research should focus on: (1) creation of vitamin–antibiotic conjugates to target a broader range of pathogens; (2) optimizing structure to ensure stability in circulation while enabling efficient intracellular drug release; (3) developing vitamin–nanoparticle conjugates for multimodal therapy (e.g., combined photothermal and chemotherapeutic approaches); and (4) exploring species-specific differences in vitamin transport to achieve selective targeting of pathogenic bacteria while preserving beneficial microbiota.

#### 2.2.2. Surface Receptor-Based “Trojan” Horses

Targeting specific surface proteins or receptors on bacterial cells forms the basis of the THS. In this approach, a carrier molecule mimics a harmless or even beneficial ligand for the bacterial cell, enabling it to bind with high specificity to a particular surface receptor. This interaction serves a dual purpose: either the targeted delivery of a lethal payload (such as an antibiotic or toxin) into the cell, or the triggering of signaling cascades that ultimately lead to pathogen death. The use of surface receptors as targets offers significant potential for overcoming antimicrobial resistance, as such precise, receptor-mediated mechanisms are more difficult for bacteria to circumvent through non-specific resistance pathways. We examined four main classes of compounds that act as such vectors: antimicrobial peptides, which possess membrane-active properties; lectins, which are specific to carbohydrate determinants on the bacterial surface; antibodies, which provide the highest binding specificity; and bacteriophage receptor-binding proteins, which are evolutionarily “tuned” to recognize specific host receptors.

##### Antimicrobial and Other Peptides

Peptides are increasingly recognized as versatile platforms for targeted antibacterial delivery. Within this category, antimicrobial peptides (AMPs) have attracted considerable attention due to their intrinsic bactericidal activity. Over 3900 AMPs have been identified to date, typically consisting of 10–50 amino acids with cationic and amphiphilic properties that enable interaction with bacterial membranes [259,260]. The antibacterial action of AMPs involves either direct membrane disruption or inhibition of intracellular processes following translocation [261]. Importantly, the selectivity of AMPs for bacterial over eukaryotic cells is not absolute, though bacterial membranes carry a higher negative charge due to acidic phospholipids, such as phosphatidylglycerol and cardiolipin [262]. Nevertheless, the specificity of AMPs also depends on many of their other structural features [263], which necessitates their chemical modification—for example, the introduction of D-amino acids, fluorinated amino acids, and other unusual amino acids into peptides to improve their selective action against pathogens [262].

The use of various AMPs as carriers and the development of hybrid AMP–antibiotic compounds have led to the synthesis of conjugates such as those with chloramphenicol [264], neomycin B [265], kanamycin [266], gentamicin [267], ciprofloxacin [268], levofloxacin [269], and vancomycin [270]. Some of them have shown better therapeutic potential relative to the free form of antibiotics (for a review, see [3]). Nevertheless, since the antibiotic carriers (AMPs) are themselves antimicrobial compounds, these conjugates cannot be classified as true Trojan Horses. This complicates the interpretation of conjugate activity and may still select for resistance, even though resistance to AMPs develops more slowly than to conventional antibiotics due to their multiple mechanisms of action [271,272,273].

An alternative approach uses peptides that are non-toxic to bacteria but capable of penetrating the bacterial cell. Cell-penetrating peptides (CPPs) represent an attractive option for delivering non-permeable antibiotics in the form of conjugates [267,274]. Most CPPs derived from the AMP family act through non-receptor-mediated pathways, interacting directly with the membrane [275].

Several examples of CPP conjugates are noted below:

(1) Tobramycin was linked at its C6″-hydroxy group via a succinate spacer to the *N*-terminus of the designed CPP Pen. The resulting conjugate, named pentobra, retained the high antibacterial activity of tobramycin. However, due to its efficient spontaneous membrane permeation, pentobra demonstrated 10^6^-fold and 10^4^-fold greater efficacy in killing *S. aureus* and *E. coli* persisters, respectively, at a concentration of 25 µM compared to tobramycin alone, while remaining non-toxic to eukaryotic cells [276]. (2) Purkayastha et al. synthesized a number of fluoroquinolones conjugated with the cell-penetrating β-peptide β^3^-h-octa-arginine. Some of the obtained conjugates demonstrated activity close to that of the free antibiotics [277]. (3) Series of chloramphenicol amides with polyamines (PAs) were synthesized. The conjugates were internalized into *E. coli* cells via the spermidine-preferential uptake system and demonstrated activity against *S. aureus* and *E. coli* [264]. Close to CPPs, some aminoamides could be considered carriers for THSs. As examples, the synthesis of conjugates based on cyclohexylalanine and arginine with nalidixic acid (NA) can be cited. In comparison to conventional NA, which has a low level of potency against *S. aureus*, these conjugates exhibited significantly improved antibacterial activity [274]. When compared to unmodified neomycin B, the prepared lysine–neomycin conjugates exhibited a 4- to 8-fold enhanced activity against the Gram-negative bacterium *P. aeruginosa*, and up to a 12-fold enhancement was observed relative to the unligated reference peptides [265].

Summarizing, peptide-based platforms offer several advantages: (1) AMPs exhibit broad-spectrum activity and slow development of resistance; (2) CPPs can transport otherwise impermeable antibiotics across bacterial membranes; and (3) the modular nature of peptides allows for chemical modification and optimization. However, limitations include: (1) AMPs themselves are antimicrobial which could enhance developing resistance; (2) selectivity for bacteria over host cells is not absolute; (3) peptides can be susceptible to proteolytic degradation; and (4) manufacturing costs may be higher than for small-molecule antibiotics. To advance peptide-based THSs, future research should focus on: (1) developing CPPs that are non-antimicrobial yet efficiently penetrate bacterial membranes, serving as true carriers; (2) developing linker to ensure payload release only within target bacteria; (3) engineering peptides with enhanced stability against proteases; and (4) exploring species-specific bacterial peptides that target unique transporters rather than relying on non-specific membrane interactions. Modern genetic engineering techniques, combined with AI-driven algorithms, now enable the design of artificial peptides with high penetrating capacity that are non-toxic to bacterial cells while exhibiting minimized proteolytic degradation and reduced toxic effects on mammalian cells. This opens up an important area for the development of hybrid molecules to combat antibiotic resistance.

##### Lectins

Lectins are carbohydrate-binding proteins that recognize specific sugar moieties on cell surfaces. In the context of THSs, lectins serve as high-precision “navigators” capable of delivering therapeutic payloads directly to pathogens, including their intracellular reservoirs—a feat unattainable with conventional antibiotics [278,279,280,281,282] (Table 17). In contrast to THS vehicles transporting into bacterial cells (see above), lectin-based approach enables rather high local concentration of the active agent precisely where bacteria are present.

Particular lectins can also be used for treating infections through targeted drug delivery, owing to their ability to specifically bind to carbohydrate structures on microbial surfaces. *P. aeruginosa* produces two well-characterized lectins, LecA (specific for galactose) and LecB (specific for fucose and mannose), which are critical for biofilm formation and virulence [288,289]. LecA- and LecB-targeted prodrugs have been developed to release antibiotics only upon interaction with these surface lectins, achieving self-destructive antibiotic release at the site of infection while minimizing systemic toxicity [283,284].

Plant lectins such as wheat germ agglutinin (WGA), which specifically binds *N*-acetylglucosamine (GlcNAc), and concanavalin A (ConA), which binds mannose residues, have been employed as targeting modules for drug delivery systems. In one study, apoferritin nanocages were functionalized with WGA and loaded with ampicillin. The resulting complex demonstrated 10-fold higher bactericidal activity against *Bacillus subtilis* compared to the free antibiotic, and remained effective in whole blood where free ampicillin was ineffective [285]. In similar work, the lectin ConA is covalently immobilized on the particle surface. The delivery of this container directly to the bacterial cell is achieved through the specific binding of the lectin ConA to polysaccharides on the *E. coli* cell wall, suggesting that in future applications, the release of an antibiotic from such a targeted carrier would ensure pathogen eradication [287].

Lectins have also been used to target intracellular bacterial reservoirs. Uropathogenic *E. coli* (UPEC) invades bladder cells via FimH lectin binding to mannose. Using plant lectins specific to GlcNAc that accumulate in the same intracellular compartments as invading UPEC, researchers proposed and proved a strategy to deliver antibiotics directly into bacterial hideouts [286].

Lectin-based strategies offer exceptional specificity due to the high affinity of lectin–carbohydrate interactions. They can target both extracellular bacteria and intracellular reservoirs, and can be engineered into diverse platforms ranging from small-molecule prodrugs to nanocarriers and even active microdevices. Beyond their use as targeting ligands, lectins have been incorporated into biomimetic delivery systems. Concanavalin A (ConA) immobilized on self-propelled micro-rockets enables active “hunting” and capture of *E. coli* cells in real time while ignoring non-target microorganisms, demonstrating the potential of lectin-functionalized microdevices for targeted antibiotic delivery [287]. In the above cited study, wheat germ agglutinin have been used as biomimetic to functionalize nanocages [285]. These biomimetic platforms—combining the natural targeting specificity of lectins with engineered nanocarriers or microdevices—expand the Trojan Horse paradigm beyond molecular conjugates to functional supramolecular systems. Limitations of the lectin applications include: (1) potential immunogenicity of plant lectins; (2) the need for multivalent presentation to achieve high avidity; (3) possible interference with host lectin functions; and (4) variability in carbohydrate expression among bacterial strains. Future research should focus on: (1) developing human-compatible lectin mimetics to reduce immunogenicity; (2) engineering multivalent lectin platforms to enhance avidity and selectivity; (3) combining lectin targeting with stimuli-responsive linkers for controlled drug release; and (4) exploring lectin-based strategies for biofilm disruption, where lectins play critical roles in maintaining biofilm architecture. The ability of lectins to navigate to intracellular compartments opens new avenues for treating persistent and recurrent infections that currently evade conventional therapy.

##### Antibodies

Antibody–antibiotic conjugates (AACs) represent a sophisticated implementation of the THS, linking a potent but potentially toxic or poorly cell-penetrating antibiotic to a monoclonal antibody that targets a specific bacterial antigen which acts as a decoy [14,21]. Following binding, the pathogen–antibody complex is taken up by host immune cells (e.g., macrophages) via phagocytosis. Within the phagolysosome, proteolytic enzymes such as cathepsins cleave the linker, releasing the active antibiotic to eliminate intracellular bacteria [14,21]. Thus, similar to lectins, application of antibody-type THSs results in the enhancement of local drug concentrations at the infection site (Table 18). Among bacterial targets, antigens such as surface proteins, lipopolysaccharides, and teichoic acids are mentioned, whilst antimicrobial payload (traditional antibiotics, antimicrobial peptides, nanoparticles) is linked to mAbs by cleavable (sensitive to the infection microenvironment conditions) or non-cleavable linkers (for review see [290]).

The most advanced AACs have been developed against *S. aureus*, including MRSA. DSTA4637A (Genentech Inc. in South San Francisco, CA, USA) is a THIOMAB™–antibiotic conjugate consisting of a monoclonal antibody targeting *S. aureus* teichoic acids linked to a rifamycin-class antibiotic (dmDNA31) via a protease-cleavable linker. In a mouse model of systemic *S. aureus* infection, a single dose of DSTA4637A (25–50 mg/kg) resulted in up to a 7.6 log_10_ reduction in bacterial load in heart, kidneys, and bones, with effects persisting for 14 days [22]. Preclinical studies in rats and primates confirmed complex but predictable pharmacokinetics [291].

An example of AAC application for targeting intracellular pathogens is the usage of the monoclonal antibody 26F8, which specifically binds to the lipopolysaccharide O-antigen of *P. aeruginosa*. It was conjugated to the arylamycin analog G2637 via a cathepsin-cleavable linker. The conjugate enabled effective intracellular killing of *P. aeruginosa* within macrophages, with the molar concentration of AAC-associated antibiotic being two orders of magnitude lower than free antibiotic required for similar efficacy against extracellular bacteria [292].

**Figure 3:**
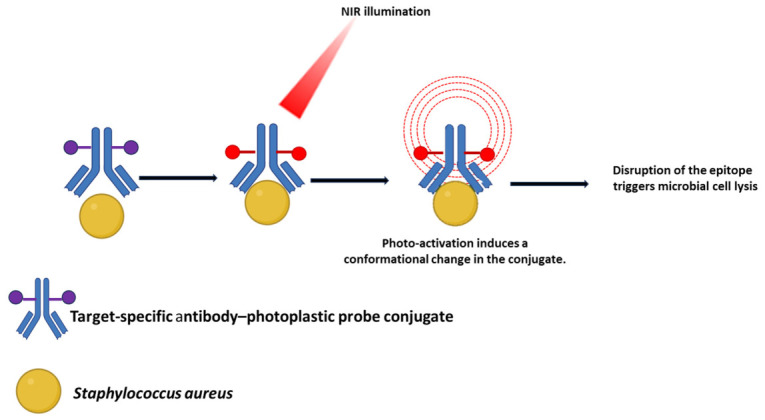
Schematic illustration of the photoimmunotechnology-based antimicrobial strategy. Upon irradiation with near-infrared (NIR) light, antibody–photoplastic probe conjugates undergo a structural transformation. This triggers epitope disruption on the microbial surface, ultimately resulting in cell lysis and pathogen inactivation. Photosensitizer color code: purple—before irradiation (inactive/ground state); red—after irradiation (activated/excited state).

Beyond traditional antibiotic payloads, antibodies have been employed to deliver photosensitizers for photodynamic therapy. The photoimmuno-antimicrobial strategy (PIAS) uses monoclonal antibodies conjugated to phthalocyanine derivatives (e.g., IRDye 700DX). Upon near-infrared irradiation, the conjugate induces physical disruption of the bacterial cell wall, enabling selective elimination of antibiotic-resistant strains without affecting surrounding cells or normal microbiota [25] (Figure 3). Similarly, a supramolecular complex combining streptavidin-labeled eosin with IgG antibodies targeting *S. aureus* protein A achieved an 8-log reduction in bacterial viability upon green light irradiation [26].

For diagnostic purposes, M13 bacteriophages have been engineered to carry both antibodies and fluorescent dyes, creating signal-amplifying probes. Anti-*S. aureus* antibodies conjugated to M13 phages loaded with Alexa Fluor 750 provided a 3.7-fold enhanced fluorescence signal at infection sites in mouse models, enabling specific detection of bacterial infections [293].

AACs offer several key advantages: (1) high specificity through antibody–antigen recognition; (2) ability to access intracellular bacterial reservoirs; (3) potential to “revive” toxic antibiotics by targeted delivery; (4) reduced systemic side effects; and (5) compatibility with diverse payloads (antibiotics, photosensitizers, imaging agents). Limitations include: (1) complex pharmacokinetics and potential immunogenicity; (2) high manufacturing costs; and (3) limited tissue penetration [290].

Future research should focus on: (1) optimizing drug-to-antibody ratios and linker chemistry for improved stability and release kinetics; (2) developing site-specific conjugation strategies to ensure batch-to-batch consistency; (3) exploring combination therapies with conventional antibiotics to overcome resistance; and (4) conducting rigorous clinical trials to translate promising preclinical candidates into approved therapies. The success of antibody–drug conjugates in oncology provides a strong foundation for the continued development of AACs for infectious diseases.

#### 2.2.3. Bacteriophage-Based “Trojan” Horses

The concept of using bacteriophages as “Trojan Horses” represents one of the most compelling strategies in modern antibacterial research. This approach repurposes bacteriophages as vehicles to deliver therapeutic cargo into bacterial cells. Unlike natural lytic phages that kill their host through replication, engineered “Trojan” phages are designed to act as precision delivery systems. Their exceptional specificity allows them to function as “smart” therapeutic agents that can analyze the cellular environment and deliver a predefined payload, such as lethal genes or CRISPR systems, only when conditions are met [294,295]. Thus, by harnessing the natural infectivity of phages while programming them to carry exogenous cargo, these bacterial viruses can be transformed into versatile molecular tools for combating pathogens, including those that are intracellular or antibiotic-resistant [296].

##### Engineering Phages

In the context of using phages as “Trojan Horses,” application of engineered phages for the targeted delivery of CRISPR-Cas systems can be used [295], which act as programmable antimicrobial agents that selectively destroy pathogens carrying antibiotic resistance genes or virulence factors. Although the use of living, replicating phages entails risks (e.g., horizontal gene transfer), the THS itself is also implemented in safer forms—through the application of phage vectors (phagemids) for nucleic acid delivery or the use of phage structures as containers for the direct injection of antibacterial molecules [295] (Table 19).

**Figure 4:**
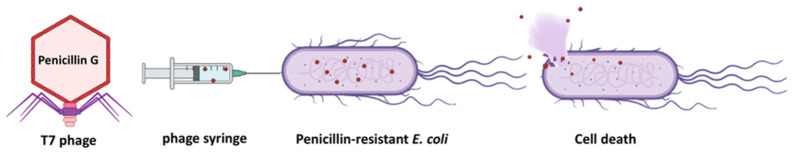
Application of phage syringe for bacterial cell inactivation. The T7 phage genome is converted into a modular format containing only structural genes and used for phage particle synthesis via a cell free protein expression system. The resulting phage syringes are loaded with penicillin G upon capsid assembly at varying antibiotic doses used as a cargo. Red dots—penicillin G.

In this regard, by constructing a modular T7 phage genome containing only structural protein genes and synthesizing it in a cell-free system, researchers achieved spontaneous encapsulation of penicillin G during capsid assembly. The resulting “phage syringes” retained the ability to specifically infect *E. coli* and inject their contents into the cytoplasm, overcoming β-lactamase-mediated resistance and effectively eradicating penicillin-resistant bacteria [297] (Figure 4).

As an example of this strategy, a system consisting of three components was developed: (1) the phage as a high-capacity carrier (with thousands of coat protein copies), (2) an antibiotic molecule (chloramphenicol) converted into a prodrug by attachment via a labile ester bond, and (3) a targeting module on the phage surface for recognition of *S. aureus* [300]. The key result was that phages loaded with chloramphenicol inhibited bacterial growth as effectively as a 10–20-fold higher concentration of the free antibiotic, demonstrating a local concentration effect [300]. This approach could also cause a “revival” of toxic or non-selective antibiotics by converting them into safe prodrugs activated only in the pathogen’s vicinity.

**Figure 5:**
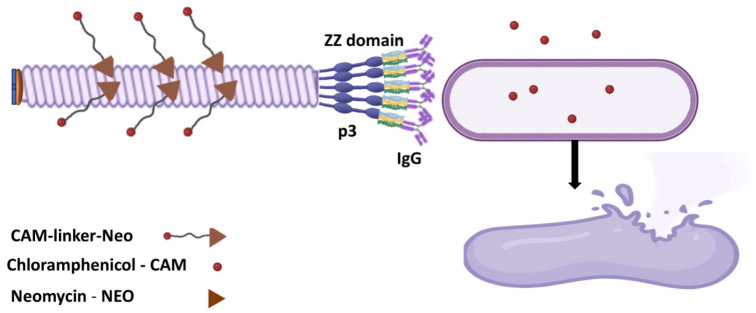
A schematic representation of the filamentous M13 bacteriophage as a drug delivery vehicle targeting pathogenic bacteria. Target specificity is achieved using antibodies that recognize the bacteria. The linkage between the antibodies and the phages is mediated by an IgG-binding ZZ-domain located on the minor coat protein of the phage. The therapeutic agent employed is chloramphenicol conjugated to neomycin via an ester bond that is cleavable by esterase.

Filamentous bacteriophages (e.g., M13, fd) have been engineered as high-capacity carriers for antibiotic delivery. A key innovation addressed the limitation of phage cargo capacity due to antibiotic hydrophobicity. Using aminoglycoside antibiotics (e.g., neomycin) as soluble branched linkers, researchers attached multiple chloramphenicol molecules to phage coat proteins, achieving up to 40,000 antibiotic molecules (chloramphenicol) per phage while maintaining structural integrity. Targeting was achieved via the ZZ domain on the phage surface, which binds bacteria-specific antibodies. This system demonstrated approximately 20,000-fold enhanced antibiotic efficacy against *S. aureus*, *Streptococcus pyogenes*, and *E. coli* compared to free chloramphenicol [298] (Figure 5).

Beyond genetic phage display, filamentous bacteriophages (e.g., M13, fd) can be chemically modified to display diverse payloads on their surface. This chemical conjugation approach has been exploited for three main applications: (1) imaging—phages conjugated with fluorophores, radioisotopes, or MRI contrast agents (“magnetophores”); (2) gene delivery—hybrid “phage–AAV” vectors combining adeno-associated virus genomes with phage capsids for enhanced transduction; and (3) drug delivery—high-capacity carriers displaying therapeutic payloads via soluble linkers [301,302]. It is important to distinguish this chemical surface engineering approach from the classical genetic phage display technology, which involves the genetic fusion of peptide or protein libraries to phage coat proteins for selection of binding ligands. Both strategies exploit the phage surface, but through fundamentally different mechanisms.

Chemical modification of phages for drug delivery paradoxically improves their safety profile. Conjugation with antibiotics via aminoglycoside linkers completely abolishes phage infectivity (a biosafety advantage) and significantly reduces immunogenicity. In mice, high doses of drug-loaded phages showed no signs of toxicity, and anti-phage antibody levels were 4–10 times lower than in mice injected with unmodified phages. Furthermore, conjugation increased circulation time in blood [299].

Phage-based THSs offer unique advantages: (1) exceptional specificity through evolutionarily refined host recognition; (2) high cargo capacity; (3) modularity for diverse payloads (antibiotics, photosensitizers, CRISPR-Cas, imaging agents); (4) potential for combination with antibodies or peptides for expanded targeting; and (5) improved safety profile upon chemical modification. Limitations include: (1) potential immunogenicity despite reduction upon modification; (2) regulatory challenges for live or modified phage therapeutics; (3) manufacturing complexity and scalability; (4) risk of horizontal gene transfer with replicating phages; and (5) limited tissue penetration for systemic infections. Future research should focus on: (1) developing new payloads to include next-generation antibiotics, CRISPR-Cas systems, and combination therapies; (2) optimizing structures for stable circulation and efficient intracellular release; (3) developing non-replicating “phage syringe” platforms for improved safety; (4) exploring phage engineering for biofilm penetration and intracellular bacterial reservoirs; (5) advancing in vivo validation in clinically relevant infection models; and (6) addressing regulatory pathways for phage-based nanomedicines.

##### Phage-Derived Endolysins 

Endolysins are enzymes produced by bacteriophages during the late stages of infection to degrade the bacterial cell wall from within, enabling release of newly formed phage particles [23,303,304]. These enzymes have attracted significant interest as antimicrobial agents because their lytic activity can be manifested upon exogenous application, causing rapid cell lysis without the need for other phage components [303].

Endolysins from phages of Gram-positive bacteria typically consist of an *N*-terminal catalytic domain and C-terminal specific cell wall-binding domains (CBDs) separated by a short linker [304]. The CBD confers species- or genus-level specificity by recognizing unique epitopes on the bacterial cell wall, making it an ideal “guidance system” for delivering various payloads to target bacteria for implementing THSs (Table 20).

Such possibility has been implemented by constructing a conjugate of the catalytic domain of an *E. coli* phage endolysin (lysep3) with the CBD from a *Bacillus amyloliquefaciens* phage endolysin (D8) as a hybrid protein (lysep3-D8) capable of killing not only *E. coli* but also *P. aeruginosa* and *Acinetobacter baumannii*. The D8 domain binds to lipopolysaccharides, disrupting outer membrane integrity and enabling the catalytic domain of lysep3 to reach the peptidoglycan [24]. The receptor-binding protein Pb5 from phage T5, which specifically binds to the outer membrane protein FhuA on *E. coli*, was fused to an endolysin catalytic domain to create “Innolysin” Ec21. This hybrid demonstrated significant bactericidal activity against clinically relevant *E. coli* strains resistant to third-generation cephalosporins [305]. “Lysocins” combine the receptor binding domain of the bacteriocin (pyocin S2) with an endolysin (GN4). The pyocin component binds to the FpvAI receptor on *P. aeruginosa* and utilizes the Ton-dependent import system to transport the endolysin across the outer membrane. Once in the periplasm, the endolysin degrades peptidoglycan, killing the cell from within. This approach demonstrated high efficacy in serum, biofilm disruption, and protection against lethal infection in mice [306].

Domain shuffling has also been used to create chimeric lysins with improved properties against Gram-positive pathogens. ClyS, a chimeric lysin active against *S. aureus* (including MRSA), was constructed by replacing the CBD of one endolysin with that from another phage, resulting in enhanced activity while maintaining species specificity [307]. ClyF, another chimeric lysin combining the catalytic domain from Ply187 and the CBD from PlySs2, demonstrated potent activity against both planktonic and biofilm forms of MRSA, with enhanced stability in complex biological environments [308].

The high specificity of endolysin CBDs has been exploited for targeted delivery of diverse payloads. CBDs from 12 different *Listeria* phage endolysins were fused to fluorescent proteins of various colors (BFP, CFP, YFP, RedStar), enabling simultaneous identification and differentiation of *Listeria serovars* in mixed cultures (multiplex assay). Magnetic particles coated with broad-specificity CBDs facilitated isolation from contaminated food samples [310]. Similarly, the CBD of CTP1L endolysin from phage ΦCTP1 was fused to GFP, enabling visualization of *Clostridium tyrobutyricum* within cheese matrices [309]. The CBD3 domain of a phage endolysin was conjugated to the photosensitizer IRDye 700DX. This conjugate specifically bound to *S. aureus* (including MRSA) and *S. epidermidis*, and upon red light activation, generated reactive oxygen species that killed planktonic bacteria and disrupted biofilms. Remarkably, the conjugate penetrated HeLa cells, retained the ability to bind intracellular bacteria, and selectively killed intracellular *S. aureus* without damaging uninfected host cells [27].

Due to their modular structure, particularly the presence of highly specific CBDs, bacteriophage endolysins have become a versatile platform for implementing the THS against bacteria. Endolysin-based THSs offer several advantages: (1) high specificity through CBD-mediated targeting; (2) modularity enabling diverse payloads (catalytic domains, fluorescent labels, photosensitizers); (3) low likelihood of resistance development due to targeting essential cell wall structures; and (4) ability to access intracellular bacterial reservoirs. Limitations include: (1) the Gram-negative outer membrane barrier requiring engineering solutions; (2) potential immunogenicity of protein-based therapeutics; (3) stability and formulation challenges; and (4) limited host range of natural CBDs, requiring engineering for broader coverage. Future research should focus on: (1) finding new CBDs to cover a broader range of pathogens, including emerging drug-resistant strains; (2) developing robust platforms for CBD-mediated delivery of non-protein payloads; (3) optimizing fusion constructs for enhanced stability, solubility, and activity in vivo; (4) exploring combination strategies with conventional antibiotics or membrane permeabilizers; (5) advancing clinical translation through rigorous toxicology and efficacy studies; and (6) engineering endolysins for biofilm penetration and eradication of persister cells.

#### 2.2.4. Bacterial Enzymes for Drug Activation (Prodrugs)

Prodrug strategies exploit bacterial enzymes to convert inactive precursors into active therapeutic agents specifically within the bacterial cell [311]. Thus, the active antibiotic is chemically modified (“masked”) to create an inactive prodrug. This masking may improve solubility, stability, or bioavailability. The prodrug is designed to be a substrate for a bacterial enzyme that is absent or significantly different in humans. Upon encountering the target enzyme, the prodrug undergoes cleavage or transformation, releasing the active antibiotic specifically within the pathogen. In our opinion, this approach could be included in the implementation of THSs as prodrugs are not pathogenic for bacteria *per se* and prodrug activating enzymes are unique to bacteria and absent in humans to prevent premature activation and systemic toxicity. At the same time, prodrugs differ from “classic players” of THSs based on specific transport or binding mechanisms. Prodrug application strategies are of significant current interest and are used for a variety of bacterial species, including pathogenic ones (for review, see, e.g., [312]). Below, we mention the most interesting examples, based on different mechanisms and experimental studies (Table 21).

In particular, β-lactamase was used as an activating enzyme: a cephalosporin scaffold linked to ciprofloxacin via an ester bond creates a prodrug that is inactive until hydrolyzed by β-lactamase. The enzyme cleaves the β-lactam ring, triggering release of active ciprofloxacin. This prodrug selectively killed clinical isolates of *E. coli* expressing various β-lactamases (CTX-M, NDM, KPC) with efficacy comparable to free ciprofloxacin, while having no effect on bacteria lacking the enzyme [35]. This approach not only activates the drug at the target site but also helps preserve the microbiota and minimize side effects.

Nitroreductases (NTRs) are enzymes that reduce nitro groups and are present in many bacteria, including mycobacteria. A 2-nitrothiazole ester-modified moxifloxacin prodrug enters persister cells (drug-tolerant non-replicators) of *M. tuberculosis*, where NTR reduces the nitro group, releasing active moxifloxacin and killing dormant subpopulations that prolong tuberculosis therapy [313]. Mycobacterial nitroreductases (Ddn, NfnB, DprE1, and others) also activate antituberculosis prodrugs such as pretomanid and delamanid. This selective reduction occurs predominantly in mycobacteria due to enzyme specificity and unique cofactors (e.g., F420), ensuring targeted delivery [314]. Piperazine diazeniumdiolates protected with a nitroaromatic group serve as nitric oxide (NO) releasing prodrugs. In the presence of *E. coli* nitroreductase NfsB, enzymatic reduction triggers a cascade leading to NO release—a potent antimicrobial agent. The prodrug reduced *E. coli* viability by up to 94%, demonstrating that the bacterium activates the toxic compound through its own enzyme [315]. Glyoxalase II (GloB) in *Staphylococcus* species (including MRSA) activates pivaloyloxymethyl (POM) ester prodrugs such as POM-ERJ, a phosphonate inhibitor of the isoprenoid biosynthesis enzyme DXR. GloB, a metallo-β-lactamase superfamily member, cleaves the ester bond, enabling cell-impermeable antibiotics to penetrate via increased lipophilicity and activate intracellularly. Mutants lacking GloB show 10–50-fold resistance, confirming enzyme specificity [316].

In order to find best prodrugs for selective activation by enzymes presented in both bacterial and human cells, comparative experimental studies should be performed. Thus, mycobacterial esterases have been investigated for activation of benzoate esters as prodrugs. Studies comparing hydrolysis rates in mycobacteria versus human plasma and rat liver homogenate allowed identification of prodrugs with selective activation in mycobacterial cells, minimizing systemic toxicity [317].

Enzyme-activated prodrug strategies offer several advantages: (1) high selectivity through exploitation of bacterial enzymes absent or distinct in humans; (2) ability to overcome permeability-based resistance; (3) potential to target persister and dormant subpopulations; (4) reduced systemic toxicity; and (5) preservation of the host microbiome. Limitations include: (1) the need for selection of enzymes with high substrate specificity to avoid off-target activation; (2) potential for resistance through mutations in the activating enzyme or transport systems; (3) variable enzyme expression levels among strains and growth conditions; and (4) complex pharmacokinetics requiring optimization of prodrug stability and activation kinetics. Future research should focus on: (1) identifying new bacterial enzymes exploited for prodrug activation, including species-specific enzymes for targeted therapy; (2) developing high-throughput screening platforms to identify optimal enzyme–substrate pairs; (3) improving linker structure to balance stability in circulation with efficient enzymatic cleavage; (4) exploring combination prodrugs that release multiple active agents upon activation; (5) investigating prodrug strategies for biofilm-associated infections, where enzyme expression may differ from planktonic bacteria; and (6) advancing clinical translation of lead candidates, particularly for tuberculosis and other chronic infections where persister populations drive treatment failure. The successful repurposing of resistance enzymes (e.g., β-lactamases) as activation mechanisms represents a particularly elegant approach to turning bacterial defenses against themselves.

#### 2.2.5. Extracellular Vesicles

Extracellular vesicles (EVs) are naturally occurring nanoparticles released by both bacterial and eukaryotic cells. Bacterial EVs (BEVs) range from 20 to 400 nm in diameter and carry diverse cargo, including proteins, lipids, nucleic acids, and metabolites [318,319]. In the context of the THS, EVs offer unique advantages as natural nanocarriers: (1) they can cross biological barriers; (2) they fuse with target cell membranes for intracellular delivery; (3) they can carry complex payloads; and (4) they exhibit intrinsic tropism for parent bacterial species [318,320,321] (Table 22).

Interestingly, human neutrophils produce antibacterial EVs loaded with cathepsin G, azurocidin, and other potent proteins that directly target and kill *S. aureus*. Similarly, plant hosts deploy EVs containing small RNAs that are taken up by attacking fungi (e.g., *Botrytis cinerea*), where they silence essential virulence genes—a natural example of cross-kingdom EV-mediated Trojan Horse interference [322].

Another example of an evolutionarily refined natural Trojan Horse is *Streptomyces* strains that package specialized metabolites (actinorhodin, candidicin, anthracyclines, actinomycin) into EVs. These vesicles fuse with target cell membranes, delivering cargo directly into the cytoplasm rather than relying on diffusion of dissolved molecules. The antimicrobial activity of the vesicles depends entirely on packaged metabolites—knocking out synthesis genes renders vesicles inactive. This system delivers lethal payloads to pathogenic bacteria (*S. aureus*, *K. pneumoniae*) and fungi (*C. albicans*, *C. neoformans*) [323].

**Table 22:** Representative extracellular vesicle-based Trojan Horse systems.

EV Source	Payload	Mechanism	Target	Key Outcome
*P. aeruginosa* (g-MVs)	Autolysins + gentamicin	Membrane fusion; combined enzymatic + antibiotic action	*E. coli*, *S. aureus*, *P. aeruginosa*	2.5× enhanced killing; overcomes permeability resistance [324]
*Streptomyces* spp.	Specialized metabolites (actinorhodin, candidicin, etc.)	Membrane fusion; cytoplasmic delivery	*S. aureus*, *K. pneumoniae*, *C. albicans*, *C. neoformans*	Direct cytoplasmic delivery; activity depends on packaged metabolites [323]
*S. aureus* (CEVs)	Ceftiofur	Natural tropism + membrane fusion; intracellular delivery	Intracellular *S. aureus*	12× enhanced killing; accelerated wound healing in vivo [32]
*S. parauberis* (SpEVs)	LL37 (antimicrobial peptide)	Natural tropism; intracellular delivery	*S. parauberis*	MIC reduced 2×; reduced peptide cytotoxicity [33]
Human neutrophils	Cathepsin G, azurocidin	Natural antibacterial EV production	*S. aureus*	Direct pathogen killing [322]
Plant (host)	Small RNAs	Cross-kingdom RNA interference	*Botrytis cinerea*	Silencing of fungal virulence genes [322]

The above “naturally emerged” application of MVs to combat pathogenic microorganisms has been successfully transferred to laboratory practice. Thus, membrane vesicles naturally released by *P. aeruginosa* (n-MVs) containing potent peptidoglycan hydrolases (autolysins) were employed. These vesicles were fused with the outer membrane of Gram-negative bacteria or attached to Gram-positive cell walls, delivering autolysins directly to the peptidoglycan and causing bacterial death. Gentamicin-induced vesicles (g-MVs) encapsulate small amounts of the antibiotic along with autolysins, creating a “combined payload.” This dual-action system killed bacteria 2.5 times more effectively than an equivalent concentration of the free antibiotic and overcame permeability-based resistance in aminoglycoside-resistant *P. aeruginosa* strains [324].

*S. aureus*-derived EVs loaded with the antibiotic ceftiofur (CEVs) efficiently penetrated infected epithelial cells due to their natural affinity for parent bacterial cells and ability to fuse with membranes. The construct delivered antibiotic directly to compartments where bacteria hide, achieving 12-fold more effective killing of intracellular bacteria compared to the free antibiotic. In a mouse model of infected skin wounds, a hydrogel containing CEVs (CEVH) accelerated healing, reduced bacterial load, and modulated inflammation [32]. Similarly, EVs from the Gram-positive fish pathogen *Streptococcus parauberis* (SpEVs) were loaded with the antimicrobial peptide LL37. SpEVs preserved carrier integrity, enhanced stability, and efficiently delivered LL37 to parent bacterial cells. Intracellular delivery increased membrane permeability and reactive oxygen species generation, reducing the MIC from 10 to 5 μg/mL compared to the free peptide, while encapsulation reduced LL37 cytotoxicity to mammalian cells [33].

Researchers have engineered “EV–mimetic” nanoparticles by coating synthetic liposomes or polymeric nanoparticles with EV membranes or with specific EV surface proteins. These hybrid systems combine the scalability of synthetic carriers with the natural targeting and immune-evasive properties of EVs. For example, macrophage-derived EV-mimetic nanoparticles have been developed to target intracellular *S. aureus* reservoirs, achieving similar efficacy to natural EVs but with greater batch-to-batch consistency [325,326]. The natural occurrence of EVs as interbacterial communication and defense vehicles—as seen in *Streptomyces* EVs delivering specialized metabolites [323]—underscores the evolutionary refinement of this THS and provides design principles for synthetic mimics.

Contemporary research convincingly demonstrates that EVs represent a versatile and highly effective platform for implementing the THS in the fight against bacterial infections. EV-based THSs offer unique advantages: (1) natural nanocarriers with inherent stability and biocompatibility; (2) ability to cross biological barriers (cell walls, eukaryotic membranes); (3) capacity for synergistic combination payloads (enzymes + antibiotics); (4) intrinsic tropism for parent bacterial species; (5) potential for engineering with heterologous cargo; and (6) ability to target intracellular bacterial reservoirs. EVs also inspire the development of synthetic biomimetic systems. Limitations include: (1) heterogeneity in size, cargo, and targeting efficiency; (2) challenges in large-scale production and purification; (3) potential immunogenicity of bacterial EVs; (4) limited understanding of in vivo biodistribution and pharmacokinetics; and (5) risk of carrying virulence factors or pro-inflammatory molecules. Future research should focus on: (1) developing scalable production and purification methods for clinical-grade EV preparations; (2) engineering EVs with heterologous and targeting moieties; (3) optimizing loading strategies to maximize payload capacity while preserving EV integrity; (4) investigating the immunogenicity of bacterial EVs and developing strategies to mitigate adverse responses; (5) exploring combination therapies using EV-delivered payloads with conventional antibiotics; (6) advancing in vivo validation in clinically relevant infection models; and (7) elucidating the mechanisms of EV-mediated cargo delivery to enable rational engineering.

### 2.3. Application of Trojan Horse Strategies Across Infection Types

The versatility of THSs enables their adaptation to diverse infection contexts. Below we discuss how specific THS designs address the unique challenges of different infection types, from systemic dissemination to localized biofilms and intracellular reservoirs.

Systemic infections (sepsis, bacteremia) require conjugates with favorable pharmacokinetic profiles—sufficient circulation time to reach distant infection sites, but not so prolonged as to cause accumulation toxicity. Small-molecule metabolite-based mimics (siderophores, vitamin B12 conjugates) are particularly suited for systemic administration due to their low molecular weight (typically <2000 Da) and favorable tissue penetration. Cefiderocol (siderophore–cephalosporin) is approved for complicated urinary tract infections and hospital-acquired pneumonia, including cases with bacteremia [40,65]. B12-ampicillin conjugates demonstrate enhanced activity against systemic *E. coli* and *Salmonella* infections in murine models [243].

Localized infections (wounds, diabetic ulcers, pneumonia) benefit from platforms that can be administered topically or via inhalation, achieving high local drug concentrations while minimizing systemic exposure. Supramolecular carriers (hydrogels, nanoparticles, inhalable microparticles) are particularly advantageous. Among of them, lactose-modified azocalixarene nanoparticles loaded with ciprofloxacin accelerated wound healing and reduced inflammation in diabetic mouse models [11]; lactose-coated nanoparticles loaded with *N*-acetylcysteine have been formulated for inhalation, achieving deep lung delivery [34]; spray-dried lactose–leucine microparticles containing anti-tuberculosis nanocarriers were effective in the lower respiratory tract [143]. Other nanocarriers could be applicable for curing localized infections: cellulose-based carriers (Section 2.2.1), ROS-responsive maltodextrin nanoparticles, and mesoporous silica nanoparticles functionalized with sugars (Section 2.2.1).

Intracellular infections (tuberculosis, salmonellosis, intracellular *S. aureus*) associated with pathogens that reside within host cells (macrophages, epithelial cells) present a unique challenge: the antibiotic must penetrate both bacterial and host cell membranes. Platforms that exploit host cell uptake mechanisms are essential. Mannosylated nanoparticles exploit macrophage mannose receptors (CD206) for targeted delivery of rifampicin and rifapentine to *M. tuberculosis*-infected macrophages, demonstrating higher activity in comparison with the free drug [164,165,166]. AACs such as DSTA4637A recognized *S. aureus* and released rifamycin intracellularly, following Fc receptor-mediated phagocytosis. As a result, a significant reduction in bacterial load in the heart, kidneys, and bones was achieved in mouse models [22,327]. Trehalose conjugates enable photodynamic killing of intracellular *M. tuberculosis* within macrophages [157,159].

Biofilm-associated infections (catheter-related infections, cystic fibrosis lung infections) present a challenge linked with biofilms imposing physical (extracellular polymeric substance matrix) and metabolic (nutrient gradients, persister cells) barriers. Platforms that penetrate biofilms or target biofilm-specific structures are required. In this regard, application of phage-derived endolysins directly degraded the *S.aureus* biofilm matrix [307,308], or the CBD3 domain of a phage endolysin conjugated to the photosensitizer disrupted biofilm architecture upon red light activation [27]. Similarly, biofilms can be disrupted by lectins targeting *P. aeruginosa* while delivering antibiotic payloads [283,284], or by rhamnose-binding proteins dispersing mature *P. aeruginosa* biofilms by sensitizing biofilms to conventional antibiotics [94].

Gastrointestinal infections require conjugates that survive passage through the stomach and small intestine, resist digestive enzymes, and release payload specifically in the colonic environment. Prodrug strategies exploiting bacterial enzymes (e.g., β-glucuronidase, nitroreductase) are particularly attractive [178,193,221].

Thus, while the core Trojan Horse principle remains constant, platform selection—whether small-molecule conjugate, nanoparticle, antibody, phage-derived protein, or prodrug—must be guided by the specific demands of the infection type and the target pathogen’s biology.

## 3. Key Design Principles for Trojan Horse Systems

The diverse experimental systems reviewed above reveal recurring design parameters that critically determine the success or failure of Trojan Horse approaches. Understanding these principles is essential for rational development of next-generation targeted antibacterial therapies. Below we synthesize the key considerations across four interconnected domains: transporter specificity, linker chemistry, activation mechanisms, and pharmacokinetic optimization.

The choice of bacterial transport system or surface receptor defines the selectivity and vulnerability profile of any Trojan Horse conjugate. Successful implementation of the THS requires that the selected uptake pathway meets several criteria: essentiality and expression during infection; bacterial specificity versus host cross-reactivity; and resistance vulnerability. The ideal choice is transporters for nutrients that are indispensable for bacterial survival under infection-relevant conditions (e.g., iron limitation, nutrient scarcity). Siderophore transporters exemplify this principle: they are upregulated during infection when free iron is scarce, ensuring conjugate uptake precisely when and where it is needed [19,42,43,44]. Conversely, transporters for sugars that are abundant in the host environment may be downregulated by catabolite repression, limiting conjugate efficacy [97,98,102]. The ideal carrier is recognized exclusively by bacterial systems with no mammalian homologs. Trehalose, rhamnose, and D-amino acids exemplify this principle: they are either absent in mammals or utilized through fundamentally different pathways [147,328,329]. However, even highly specific carriers may interact with host proteins at high concentrations, necessitating careful dose optimization. Conjugates that rely on a single transporter are vulnerable to resistance through transporter mutations. The clinical experience with cefiderocol is instructive: while highly effective initially, resistance can emerge via mutations in TonB-dependent transporters or siderophore uptake systems [47,51]. Strategies to mitigate this vulnerability include: (i) application of essential transporters that cannot be easily lost; (ii) designing conjugates that utilize multiple parallel uptake pathways; and (iii) combining Trojan Horse agents with conventional antibiotics that engage different targets.

**Figure 6:**
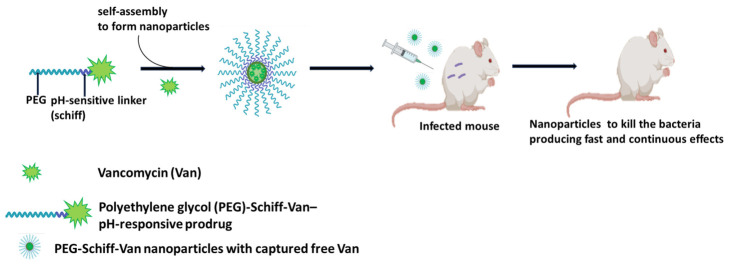
pH-responsive PEG–vancomycin conjugate nanoparticles with captured free vancomycin maximize antibacterial activity via controlled antibiotic release in acidic microenvironments.

The linker connecting a carrier to the payload is arguably the most critical component determining conjugate performance. Its design must resolve a fundamental paradox: the linker must be sufficiently stable to survive systemic circulation, yet efficiently cleaved upon reaching the target to release the active drug. The choice depends on the subcellular localization of the drug target and the mechanism of payload release. For targets located in the periplasm (e.g., penicillin-binding proteins in Gram-negative bacteria), non-cleavable linkers may suffice, as the conjugate can exert activity without intracellular release [40]. For cytoplasmic targets, cleavable linkers are essential. Common cleavable motifs include: enzymatically cleavable linkers (esters, amides, and peptides hydrolyzed by bacterial esterases, β-lactamases, or cathepsins (for intracellular delivery)) [35,78,317]; redox-sensitive linkers (disulfide bonds that are reduced by intracellular glutathione, enabling drug release in the reducing environment of the bacterial cytoplasm) [74,114]; and pH-sensitive linkers (acetal or hydrazone bonds that hydrolyze under acidic conditions encountered in phagolysosomes or infection microenvironments) [9,10,11] (Figure 6). The cephalosporin-based linker exemplifies elegant design: it is stable in circulation but selectively cleaved by bacterial β-lactamases, turning a resistance mechanism into an activation switch [35].

Premature linker cleavage leads to off-target toxicity, reduced efficacy, and poor pharmacokinetics. The success of cefiderocol is partly attributable to its optimized linker, which resists hydrolysis in plasma while allowing efficient drug release upon bacterial uptake [65,70]. Preclinical evaluation must include rigorous assessment of linker stability in plasma, liver homogenates, and simulated physiological conditions.

For conjugates that must release the active drug inside bacterial cells, the activation mechanism must be reliably triggered in the target compartment. Bacterial enzymes offer high specificity for prodrug activation. β-Lactamases, nitroreductases, and esterases have all been successfully exploited [35,313,315,317]. A key advantage is that these enzymes are often upregulated in resistant strains, creating a “self-destructive” mechanism. However, enzyme expression can vary with growth phase and environmental conditions, limiting the expected performance in vivo.

For supramolecular carriers (antibodies, extracellular vesicles) that are internalized by host cells, activation relies on host proteases (e.g., cathepsins) within phagolysosomes [14,21,22]. This strategy is particularly effective for targeting intracellular pathogens such as *M. tuberculosis* and *S. aureus* persisters that reside within macrophages. The linker must be designed to resist cleavage in circulation while remaining susceptible to lysosomal proteases.

Hypoxia, acidic pH, and elevated reactive oxygen species characteristic of infected tissues could be exploited for a linker cleavage and controlled release. The lactose–azocalixarene system (LacAC4A) exemplifies this approach: bacterial azoreductases in hypoxic wound environments trigger nanoparticle disassembly and antibiotic release [9,10,11].

Even the most elegantly designed conjugate will fail if its pharmacokinetic properties are suboptimal. Protein-based carriers (antibodies, phages, endolysins, lectins) elicit immune responses that can neutralize efficacy, cause adverse reactions, or limit repeat dosing. Strategies to mitigate immunogenicity include PEGylation, humanization of antibody fragments, and chemical modification that reduces immunogenic epitopes [22,291,299]. Notably, conjugation of antibiotics to filamentous phages via aminoglycoside linkers not only preserves cargo capacity but also reduces anti-phage antibody responses compared to unmodified phages [299].

The size and physicochemical properties of the conjugate determine its ability to reach infection sites. Small-molecule siderophore conjugates (Mw < 2000 Da) generally penetrate tissues efficiently and can achieve high bioavailability. Supramolecular carriers (antibodies, phages, EVs) face greater barriers to tissue penetration but offer advantages in targeting specificity and payload capacity. Local administration (topical, inhaled, intravesical) may circumvent systemic delivery challenges for certain applications [9,10,11,34,143].

Conjugate design must consider potential toxicity of both carrier and payload. Ideally, the carrier itself is non-toxic and biocompatible (e.g., sugars, vitamins, endogenous metabolites). For carriers with intrinsic biological activity (e.g., antimicrobial peptides, antibodies), careful selection is required to avoid unintended effects. The prodrug approach offers an additional safety layer: the antibiotic should remain inactive until activated by bacterial enzymes for reduction in systemic toxicity [14,35,311,312,313].

For antibody–antibiotic conjugates, the drug-to-antibody ratio (DAR) critically influences both efficacy and toxicity. High DARs may improve potency but can compromise antibody stability, alter pharmacokinetics, and increase immunogenicity. Site-specific conjugation strategies (cysteine-based, non-canonical amino acids) enable precise control of the DAR and batch-to-batch consistency [22,290].

Conjugation can alter the pharmacophore’s conformation, charge distribution, or steric accessibility, potentially reducing intrinsic activity. Successful designs employ linkers that attach at positions not critical for target binding. For example, cephalosporins tolerate modification at the C-7 position without losing β-lactamase stability or target affinity, making them ideal scaffolds for siderophore conjugation [65,70]. Similarly, aminoglycosides can be modified at the 5″-hydroxy position without compromising ribosomal binding [276].

Unlike antibiotics, photosensitizers (PSs) can function while covalently attached to the carrier, making non-cleavable linkers preferred to simplify design and avoid off-target release [12,25,27,133]. However, the linker must maintain sufficient flexibility to allow the PS to interact with molecular oxygen; rigid linkers may reduce quantum yield. The short diffusion distance of ROS (~0.1 μm) requires PS localization within this range of target structures. For Gram-negative bacteria, the outer membrane necessitates delivery to the periplasm or attachment to carriers that disrupt membrane integrity [80,157]. Lectins, antibodies, and phage-derived cell-binding domains provide high-affinity surface binding without active transport [27,133,283,284], while trehalose conjugates exploit mycobacterial-specific transporters to deliver PSs across the complex cell envelope [157,159]. Many PSs possess intrinsic fluorescence, enabling theranostic applications where the same conjugate serves both detection and therapy, as exemplified by galactose–BODIPY conjugates (GalTEBB-1, GalTEBB-2) [133].

Fluorescent probes for bacterial detection represent a rapidly growing application of the Trojan Horse concept. Unlike therapeutic payloads, diagnostic probes demand high signal-to-noise ratio, rapid turnover, and minimal perturbation of bacterial physiology. Design of fluorescent probes is based on diverse mechanisms to generate detectable signals. Turn-on probes operate as follows: the fluorophore is quenched in the conjugate but becomes fluorescent upon enzymatic cleavage or metabolic incorporation. The DMN-Tre probe exemplifies this: it is non-fluorescent until incorporated into mycobacterial cell wall lipids, whereupon it fluoresces, enabling detection of live *M. tuberculosis* within macrophages [148,149,151]. Through metabolic incorporation, fluorescent D-amino acids are incorporated directly into peptidoglycan by bacterial transpeptidases, labeling the cell wall with high specificity without requiring active transport of the intact probe [241,242]. Transport-dependent accumulation causes probes that are actively transported but not metabolized to accumulate intracellularly, amplifying signal. The maltose–carbazole probe (Mal-Cz) enters *E. coli* via LamB/MalE transporters and is hydrolyzed intracellularly, generating fluorescence specifically in bacteria expressing the maltose system [120].

Diagnostic applications demand higher selectivity than therapeutics, as false positives can lead to unnecessary treatment or misdiagnosis. The ideal probe exploits a bacterial-specific transporter or metabolic pathway entirely absent from mammalian cells. Trehalose-based probes for mycobacteria achieve this: trehalose is not synthesized by mammals, and its transport systems are unique to bacteria [147,150,155]. Similarly, azide-modified sugars enable bioorthogonal labeling of anaerobic gut bacteria with minimal background [193]. For clinical diagnostics, rapid detection with high sensitivity is essential. The solvatochromic trehalose probe (3HC-3) achieves a 10-fold increase in fluorescence intensity compared to earlier probes, enabling detection within minutes rather than hours [150]. Fluorine-18-labeled disaccharides enable positron emission tomography (PET) imaging of bacterial infections with high sensitivity, distinguishing infection from sterile inflammation [194]. These advances demonstrate that optimizing signal generation mechanisms is as critical as targeting specificity.

Diagnostic probes must not alter bacterial behavior or viability, as this could confound interpretation or exacerbate infection. Metabolic probes such as fluorescent D-amino acids are incorporated at sub-inhibitory concentrations and do not disrupt cell wall integrity [241]. Similarly, azide-modified sugars are incorporated into newly synthesized macromolecules without blocking essential pathways [193]. The ability to distinguish multiple bacterial species simultaneously is valuable for diagnostic applications. Phage endolysin cell-binding domains fused to different fluorescent proteins (BFP, CFP, YFP, RedStar) enable serovar-specific detection of *Listeria* in mixed cultures, demonstrating the potential of modular carrier platforms for multiplexed diagnostics [310].

The field now stands at an inflection point where mechanistic understanding of cargo-dependent requirements must be integrated with advances in synthetic chemistry, structural biology, and pharmacokinetic modeling to realize the full potential of this strategy.

## 4. Comparative Analysis of Trojan Horse Platforms, Their Challenges and Limitations

Table 23 synthesizes the advantages, limitations, and clinical status of five major THSs: metabolite-based mimics, surface receptor-targeting systems (antibodies and lectins), bacteriophage-based systems, extracellular vesicles, and enzyme-activated prodrugs.

As shown in Table 23, different THS platforms exhibit distinct advantages and limitations. This necessitates a context-dependent selection of specific platforms for particular applications and infection types in practice.

Thus, for Gram-negative bacteria, the outer membrane presents a major barrier. Metabolite-based mimics that utilize TonB-dependent transporters (siderophores) or specific porins (maltose, cellobiose) have proven effective. Bacteriophage endolysins require engineering with receptor-binding proteins or fusion with membrane-disrupting domains to overcome the outer membrane. Extracellular vesicles from Gram-negative donors naturally cross the outer membrane via fusion. For Gram-positive bacteria, which lack an outer membrane, all platforms are viable, though antibody-based targeting of teichoic acids (e.g., DSTA4637A) and endolysins are particularly advanced.

Conversely, the nature of the infection—extracellular, intracellular, or biofilm-associated—also dictates the choice of the THS approach (as discussed in Section 2.3). Collectively, these considerations underscore the need for comprehensive comparative studies of the various strategies to enable their rational application to specific infection types and the causative pathogens.

Do different platforms select for resistance at different rates? For example, metabolite-based mimics selecting transporter mutations could be compared with endolysins targeting essential peptidoglycan structures where resistance is rare [330]. Which platform combinations are synergistic? Pairing a metabolite-based conjugate with an endolysin or AAC could potentially delay resistance emergence and enhance efficacy. Comparative studies of microbiome disruption by different platforms would be valuable. Platforms with narrow targeting (e.g., species-specific antibodies, phages) may preserve commensal bacteria better than broader-spectrum metabolite mimics.

Addressing these questions should be a priority for future research to accelerate clinical translation of THS.

There are hundreds of Trojan Horse conjugates with demonstrated in vitro activity against clinically relevant pathogens, including multidrug-resistant strains as mentioned above. However, only one such agent—cefiderocol—has received regulatory approval. This dramatic attrition between bench and bedside is not merely a reflection of normal drug development failure rates; it points to specific, recurring barriers that disproportionately affect THSs. A conservative estimate based on our analysis of the published literature suggests that fewer than 5% of reported siderophore–antibiotic conjugates have been evaluated in any in vivo infection model, and fewer than 1% have progressed to human trials. For other platforms (AACs, phage-based carriers, EVs), the fraction is even smaller. This attrition rate substantially exceeds that of conventional antibiotic discovery programs, indicating unique challenges inherent to conjugate-based strategies.

Several conjugates that showed promising in vitro and in vivo activity failed to advance. BAL30072 (siderophore monosulfactam, Basilea, Basel, Switzerland) was discontinued after Phase I due to hepatotoxicity signals. MC-1 (siderophore-monocarbam, Pfizer, Groton, CT, USA) and several GSK candidates were halted due to unfavorable pharmacokinetics (short half-life, rapid clearance) and insufficient efficacy in animal infection models. For AACs such as DSTA4637A (anti-teichoic acid–rifamycin, Genentech, South San Francisco, CA, USA), while preclinical efficacy was robust, clinical development faces challenges related to manufacturing complexity, drug-to-antibody ratio control, and potential immunogenicity upon repeat dosing for chronic infections. These examples underscore that in vitro potency is a necessary but woefully insufficient predictor of clinical success.

As detailed in Section 3, the major barriers to clinical translation include: (i) premature linker cleavage and unfavorable pharmacokinetics; (ii) immunogenicity of protein-based carriers; (iii) poor predictability of in vitro activity for infection-relevant conditions; and (iv) off-target toxicity not captured by standard cell culture assays. In particular, nephrotoxicity has been a particular challenge for conjugates that accumulate in the kidney due to transporter expression or clearance mechanisms [331].

These challenges have contributed to the high attrition rate observed across all THSs.

The success of cefiderocol offers valuable lessons. Its development involved extensive optimization of the linker (catechol–cephalosporin with a stable amide bond), careful selection of the cephalosporin core (cefiderocol retains activity against many β-lactamases), and rigorous preclinical pharmacokinetic modeling.

The in vitro–in vivo gap should not be interpreted as a failure of the THS. Rather, it highlights that in vitro potency is a necessary but woefully insufficient predictor of success. The field must adopt more rigorous, early-stage in vivo evaluation and acknowledge that translation is the central challenge, not discovery of new conjugates.

Trojan Horse conjugates that rely on active transport for cellular entry are inherently vulnerable to resistance mutations that disrupt the targeted uptake pathway. Cefiderocol, despite its clinical success, selects for resistance mutations in iron transport systems. Laboratory evolution studies have identified mutations in the TonB-dependent siderophore receptor pirA and the ferric enterobactin transporter piuA that reduce cefiderocol uptake in *P. aeruginosa* [47,51]. Similarly, resistance to the natural sideromycin albomycin emerges via mutations in the ferrichrome transporter FhuA [47,51]. The clinical experience with cefiderocol, while still favorable relative to alternatives, has documented resistance emergence during therapy, particularly in patients with prior exposure to siderophore–cephalosporins [332].

For sugar-based conjugates, mutations in ABC transporter components (e.g., MalE, MalK, or the cellobiose transporter CebE/MsiK) abrogate conjugate uptake and activity [97,161]. The essentiality of the transporter is a critical consideration: targeting transporters required for growth under infection conditions may reduce the fitness of resistant mutants, slowing resistance emergence. However, even essential transporters can tolerate loss-of-function mutations if bacteria acquire the metabolite through alternative pathways or if the infection environment provides the nutrient.

The vulnerability to transporter mutations varies across bacterial species and transporters. For *M. tuberculosis*, the trehalose-specific transporter LpqY-SugABC is essential for growth on trehalose, but laboratory mutants with reduced transporter expression can still survive on alternative carbon sources [161]. For obligate pathogens that depend on a single uptake system for an essential nutrient (e.g., *Tannerella forsythia* requires exogenous MurNAc), the resistance risk may be lower [198].

Antibodies, phage proteins, endolysins, lectins, and bacterial extracellular vesicles are all immunogenic to varying degrees. The immune response can manifest as neutralizing antibodies that block therapeutic efficacy, accelerated clearance that reduces exposure, or hypersensitivity reactions that limit repeat dosing. AACs face the same immunogenicity challenges as antibody–drug conjugates in oncology. While humanized and fully human antibodies reduce but do not eliminate anti-drug antibody responses, the conjugation process itself can create neo-epitopes. The THIOMAB™ platform used in DSTA4637A incorporates site-specific conjugation to reduce heterogeneity and potentially immunogenicity, but clinical data on anti-drug antibody formation in humans are not yet available [22,291].

Unmodified bacteriophages elicit robust anti-phage antibody responses that clear the phage within days, limiting repeat dosing. However, chemical modification of phages—particularly conjugation of antibiotics via aminoglycoside linkers—paradoxically reduces immunogenicity. In mouse studies, drug-loaded phages elicited 4- to 10-fold lower anti-phage antibody titers compared to unmodified phages, and conjugation also extended circulation time [299]. The mechanism may involve masking of immunogenic epitopes or altered phagocytosis.

Bacterial EVs carry pathogen-associated molecular patterns (PAMPs) including lipopolysaccharide, lipoteichoic acid, and peptidoglycan fragments that trigger innate immune responses via Toll-like receptors. While this adjuvant effect may enhance antimicrobial activity, it also risks inflammatory toxicity. Engineering EVs to remove or neutralize PAMPs while retaining targeting and cargo functions is an active research area [318,320].

The structure of the linker connecting carrier to payload is of particular importance for ensuring the best performance of the conjugate. In particular, the anticipated site of delivery and action of the conjugates within the bacterial cell, their stability in the host organism, and their dependence on the cargo type must be taken into account in the design of the linker, as discussed above in Section 3.

Conjugation of a small-molecule antibiotic to a carrier (siderophore, sugar, vitamin) dramatically alters its molecular weight, charge, and hydrophilicity, with unpredictable effects on absorption, distribution, metabolism, and elimination (ADME). A conjugate that shows excellent in vitro activity may have poor oral bioavailability, rapid renal clearance, or unexpected tissue accumulation. Small-molecule conjugates (Mw < 2000 Da) generally distribute well to most tissues but may be rapidly cleared. Supramolecular carriers (antibodies, phages, EVs, Mw > 50 kDa) have longer circulation times but face barriers to tissue penetration. For intracellular pathogens, this can be advantageous if the carrier is designed for uptake by macrophages or other host cells. For extracellular infections, large carriers may not reach the infection site at sufficient concentrations.

Conjugates that rely on nutrient transporters for uptake must compete with endogenous substrates. High concentrations of natural siderophores, sugars, or vitamins in the host environment or microbiota can competitively inhibit conjugate uptake. This is particularly relevant for gut pathogens, where dietary and microbial metabolites create a complex competitive landscape. At the same time, conjugates based on natural (including bacteria-unique) substrates such as sugars may be subject to degradation by the gut microbiota, thereby precluding their oral (*per os*) administration.

The four challenge areas discussed above are not independent. Transporter mutations are more likely to emerge if conjugate concentrations at the infection site are subtherapeutic due to poor pharmacokinetic or premature linker cleavage. Immunogenicity accelerates clearance, exacerbating pharmacokinetic limitations. A conjugate optimized for one parameter may perform poorly on another. Successful clinical translation therefore requires holistic optimization across all four domains, with iterative feedback between in vitro characterization, in vivo pharmacokinetic modeling, and toxicity assessment.

## 5. Conclusions

The escalating threat of antibiotic resistance, coupled with the inherent limitations of conventional broad-spectrum therapeutics, necessitates a paradigm shift in how we approach antibacterial therapy. This review has explored the transformative potential of TDD as a strategic alternative, with a particular focus on leveraging unique BTSs and other biological structures/mechanisms for realization of the THS to outmaneuver pathogens.

Bacterial transport systems represent a highly promising platform for THS development due to their specificity, efficiency, and essential role in bacterial survival. By exploiting the natural uptake pathways for siderophores, unique sugars (such as rhamnose, trehalose, and maltose), cell wall components (like peptidoglycan fragments and D-amino acids), and vitamins, therapeutic payloads can be delivered directly into the bacterial cytoplasm or periplasm. This approach enables the “revival” of antibiotics that are poorly permeable or toxic, significantly increasing their local concentration at the target site and potentiating their antimicrobial activity. By confining drug release to the bacterial cell, it minimizes systemic exposure and off-target effects on the host microbiome and tissues, a critical advantage over traditional antibiotics. The THS can bypass common resistance mechanisms, such as reduced membrane permeability and efflux pumps, by actively importing the drug conjugate through essential, high-affinity transporters. To date, a substantial number of conjugates of antibiotic compounds and reporter molecules with naturally transported substrates have been designed and synthesized. Many of these conjugates have demonstrated markedly enhanced efficacy as antibacterial and diagnostic agents compared with the corresponding unconjugated compounds. However, most of these studies have been conducted in vitro, and only a limited number have been evaluated in vivo in animal models.

At the same time, a number of prospective, naturally transported substrates (e.g., raffinose, arabinose, arabinooligosaccharides, β-glycosides) remain underexplored for THS realization.

Beyond metabolite-based mimics, supramolecular structures with high affinity for bacterial cells are utilized in realization of the THS. Bacteriophages and their derived proteins (endolysins, receptor-binding proteins) offer unparalleled specificity for bacterial recognition and can be engineered to deliver lethal CRISPR systems, antibiotics, or photosensitizers. Antibodies, antimicrobial peptides, and lectins provide high-precision targeting of surface receptors, enabling both direct killing and the delivery of conjugated therapeutics to intra- and extracellular pathogens. Extracellular vesicles as natural nanocarriers and bacterial enzymes for prodrug activation represent an evolutionarily refined and highly selective approach to drug delivery.

Despite the significant progress and promising preclinical results, the translation of THSs from bench to bedside faces formidable challenges. These include resistance via transporter mutations, immunogenicity of biological carriers, linker instability, and complex pharmacokinetics that are poorly predicted by in vitro assays. The field must confront these challenges head-on rather than treating them as afterthoughts. High-throughput genomic and proteomic screens can identify novel transporters less prone to resistance mutations. Advances in linker chemistry and protein engineering can improve stability and reduce immunogenicity. Early-stage in vivo pharmacokinetic evaluation should become standard practice, not an endpoint reserved for lead candidates.

High-throughput genomic and proteomic screens can identify novel transporters and surface receptors specific to priority pathogens, including those associated with chronic and biofilm-associated infections. Advances in linker chemistry should focus on developing stimuli-responsive systems that are stable in circulation but efficiently processed by bacterial enzymes, host proteases (for intracellular delivery), or microenvironmental signals (pH, redox, hypoxia). Protein engineering, humanization of antibody fragments, and the use of synthetic or human-compatible scaffolds (e.g., engineered protein cages, synthetic polymers) can mitigate immune responses while preserving targeting specificity. Early-stage evaluation of conjugates should incorporate rigorous pharmacokinetic and biodistribution studies to guide optimization of dosing regimens and identify potential toxicity issues before advancing to efficacy studies. Preclinical evaluation should move beyond standard planktonic cultures and acute infection models to include biofilm-associated infections, intracellular pathogen reservoirs, and chronic infection models that better recapitulate human disease. Given the potential for resistance to carrier-based delivery, combining THS platforms with conventional antibiotics or adjuvants that target alternative pathways may enhance efficacy and delay resistance emergence. For supramolecular platforms (phages, EVs, antibody conjugates), scalable manufacturing, quality control, and regulatory pathways must be established to facilitate clinical translation.

A more balanced perspective—one that acknowledges both the promising preclinical efficacy and the recurrent translational failures—is essential for the field to mature. Reporting of negative results and failed clinical trials should be encouraged to inform rational platform selection and accelerate learning.

The THS offers a powerful conceptual framework for next-generation antimicrobials. However, the field must confront an uncomfortable truth: after decades of research and hundreds of published conjugates, only one has reached patients. Closing the *in vitro–in vivo* gap will require a cultural shift—from celebrating in vitro potency as an endpoint to recognizing that translation is the central, rate-limiting challenge. This stark *in vitro–in vivo* gap is not merely a reflection of normal drug development attrition; it points to specific, recurring translational barriers—including premature linker cleavage, immunogenicity of biological carriers, unexpected off-target toxicity, and resistance via transporter mutations—that have disproportionately hindered Trojan Horse conjugates. Therefore, while the scientific foundations are strong, the clinical potential of this strategy will only be realized through a concerted, interdisciplinary effort to systematically address these limitations. Realizing this potential will require not only continued innovation in conjugate design but also a concerted effort to address the translational challenges that have limited progress to date.

These challenges, often operating synergistically, explain the striking attrition rate (estimated >99% from in vitro hits to approved drug) and underscore that in vitro potency is a necessary but woefully insufficient predictor of clinical success.

For protein-based platforms (antibodies, lectins, phage proteins), immunogenicity remains a major hurdle: neutralizing anti-drug antibodies accelerate clearance, limit repeat dosing, and can cause hypersensitivity reactions. Even “humanized” scaffolds are not immune, and chemical modification (e.g., PEGylation) offers only partial mitigation. Unlike conventional small-molecule antibiotics, Trojan Horse conjugates occupy a hybrid category—part novel chemical entity, part biologic (for protein-based carriers)—which complicates regulatory review. Specific guidance from agencies for conjugate-based antibacterials is lacking. Key challenges include: defining appropriate non-inferiority margins when the active antibiotic is already approved; demonstrating that the targeting moiety provides added clinical value beyond simple co-administration; and establishing bioequivalence criteria for linker chemistry and drug-to-carrier ratios.

Regarding the reasons for clinical trial failures of specific candidates, a recurring theme across all platforms is the poor predictability of standard in vitro assays for in vivo performance. Biofilm-associated infections, intracellular pathogen reservoirs, and nutrient-rich host environments (where transporter expression may be downregulated) are poorly modeled in routine screening. The field must adopt more rigorous, early-stage in vivo pharmacokinetic and efficacy evaluation, including infection models that recapitulate human disease complexity.

The field stands at a critical juncture: the scientific foundations are strong, but systematic, interdisciplinary efforts are needed to bridge the gap between proof-of-concept and clinical reality.

## Figures and Tables

**Table 2:** Representative cellobiose and cellulose-based Trojan Horse systems.

Platform	Delivery Mechanism	Target Pathogens	Key Outcome
Flocculosin (cellobiose lipid)	ABC transporter hijacking + outer membrane disruption	*E. coli*, *C. difficile*	8–32× MIC reduction [103]
β-Cyclodextrin–cellulose fibers	Controlled release via surface adhesion	*E. coli*, *S. aureus*	15-day sustained inhibition [30]
Protoporphyrin IX–cellulose nanofibers	Topological delivery + photodynamic activation	*E. coli*, *S. aureus*	>5 log CFU reduction (20 min illumination) [31]
Nanocellulose–selenium nanocomposite	Carrier-mediated surface delivery	*E. coli*, *P. aeruginosa*, *S. aureus*, *C. albicans*	MIC as low as 15.6 μg/mL [104]

**Table 3:** Representative maltose-based Trojan Horse systems.

Platform	Function	Target	Key Outcome
Ciprofloxacin–maltose conjugate	Antibiotic delivery	*E. coli*	5–25× enhanced activity [112]
Maltotriose–perylene (Cpd-1)	Auto-inducing delivery	*E. coli*	Crosses both membranes; induces own uptake [113]
TM-TMP (thiolmaltose–trimethoprim)	Prodrug	*E. coli*	>250× solubility; <1% serum cleavage [114]
MMCC (mannose–maltose–colistin)	Dual-targeting prodrug	*K. pneumoniae*	Reduced nephrotoxicity; efficacy vs. intracellular bacteria [115]
MDNP (maltodextrin nanoparticles)	Persister awakening	*S. aureus*	Resensitizes dormant bacteria to rifampicin [116]
Maltose–thiosemicarbazone conjugates	Targeted antibiotic	MRSA, *E. coli*	MIC 0.78 μg/mL; low cytotoxicity [117]
Mal-Cz fluorescent probe	Diagnostic beacon	*E. coli*, *S. epidermidis*	Selective detection in mixed cultures [118,119,120]

**Table 4:** Representative galactose-based Trojan Horse systems.

Platform	Targeting Strategy	Mechanism	Target	Key Outcome
Galactocluster–siderophore conjugates	LecA binding + iron uptake	Dual targeting via lectin and siderophore pathways	*P. aeruginosa*	Enhanced anti-pseudomonal activity [131]
Lactose–albumin microspheres with gentamicin	Galactose–K88 fimbriae binding	Surface adhesion + local drug release	Enterotoxigenic *E. coli*	Targeted antibiotic delivery [132]
GalTEBB-1 and GalTEBB-2	LecA/LecB targeting	Photodynamic inactivation	*P. aeruginosa*, *S. aureus*	Theranostic: imaging + bactericidal activity [133]
Tetravalent D-galactose neoglycoconjugate	Lectin blockade	Anti-adhesive + liposomal delivery platform	Pathogenic bacteria	Prevents biofilm formation [130]
Lactose azocalixarene–ciprofloxacin (LacAC4A)	LecA targeting + hypoxia-responsive release	Active targeting + triggered intracellular release	MDR *P. aeruginosa*	Enhanced efficacy; accelerated wound healing [11]
Lactose electrosprayed microparticles	Galactose-mediated attraction	Biocompatible carrier + loc	*S. aureus*, *P. aeruginosa*	Concentration-dependent bacterial killing [134]

**Table 5:** Representative raffinose-based systems relevant to Trojan Horse strategies.

Platform	Function	Mechanism	Target	Key Outcome
Raffinose in fermented milk	Prebiotic activation	Selective stimulation of *L. reuteri*; enhances reuterin production	*S. aureus* biofilms	Inhibits biofilm formation and virulence throughout storage [138]
Raffinose–drug conjugates (conceptual)	Targeted antibiotic delivery	Hijacking bacterial ABC transporters (not yet experimentally realized)	Gram-negative and Gram-positive bacteria	Proposed for future development

**Table 6:** Representative lactose-based Trojan Horse systems.

Platform	Function	Mechanism	Target	Key Outcome
Ciprofloxacin–lactose electrosprayed microparticles	Antibiotic carrier	Lactose as biocompatible matrix and bacterial attractant	*S. aureus*, *P. aeruginosa*	Concentration-dependent bacterial killing; “empty” particles stimulate growth [134]
NAC–PLGA nanoparticles with lactose coating	Pulmonary delivery	Lactose as cryoprotectant and mask for lung deposition	*M. tuberculosis*	4× increased antimycobacterial activity [34]
Lactose–leucine microparticles with anti-TB nanocarriers	Pulmonary delivery	Lactose forms porous particles for deep lung deposition	*M. tuberculosis*	Targeted delivery to alveolar macrophages [143]
LacAC4A–ciprofloxacin (lactose azocalixarene)	Smart nanosystem	LecA targeting + hypoxia-responsive azoreductase cleavage	MDR *P. aeruginosa*	Enhanced efficacy; reduced inflammation; accelerated wound healing [11]
Lactose–fatty acid esters (URB1076, URB1077)	Membrane-active conjugates	Lactose as hydrophilic carrier; fatty acids disrupt membranes	*E. coli*, *S. aureus*, *P. aeruginosa*	MIC 64–128 μg/mL; broad-spectrum activity [144]

**Table 7:** Representative trehalose-based Trojan Horse systems.

Platform	Function	Mechanism	Target	Key Outcome
DMN-Tre	Fluorescent probe	Cell wall labeling	*M. tuberculosis*	Detection in macrophages and sputum [148,149,150,151,152]
CDG-Tre (cephalosporinase-dependent green trehalose)	Fluorescent probe	Enzyme-activated labeling	*Mycobacteria*	Selectivity for Corynebacterineae [151,153,154]
Tre-Cz (trehalose–carbazole)	Fluorescent probe	Intracellular accumulation	*Mycobacteria*	Detection in mixed cultures and sputum [155]
Trehalose–Pks13 inhibitor conjugates	Antimycobacterial therapy	ABC transporter-mediated uptake	*M. smegmatis*, *M. tuberculosis*	Enhanced potency and selectivity [156]
Trehalose–BODIPY conjugate	Photodynamic inactivation	Photosensitizer delivery	*M. smegmatis*, *M. abscessus*	MIC 0.5–33 μM after illumination [157]
Trehalose–porphyrin conjugate	Photodynamic inactivation	Photosensitizer delivery	*M. smegmatis*	Growth inhibition at 10 mM after illumination [158]
TCC2Tre (trehalose–tricarbocyanine)	Photodynamic inactivation	Photosensitizer delivery via trehalose transporters	*M. smegmatis*, *M. tuberculosis*	99.9% photoinactivation at 20 μM [159]

**Table 8:** Representative mannose-based Trojan Horse systems.

Platform	Function	Mechanism	Target	Key Outcome
Mannose-coated PLGA–PEG nanoparticles with rifapentine	Macrophage-targeted delivery	Mannose receptor-mediated endocytosis	*M. tuberculosis*	4× dose reduction; 60 h sustained release [165]
Mannose-modified lipid nanoparticles	Macrophage-targeted delivery	Mannose receptor targeting	*M. tuberculosis*	Enhanced efficacy; reduced side effects [166]
MP-MENP (mannose photothermal nanoparticles)	Photothermal therapy	Monocyte/macrophage targeting + laser activation	MRSA, *S. aureus*	Complete infection eradication; immune activation [167]
Mannose-functionalized nanoparticles	Bacterial adhesion blockade	FimH binding	*E. coli*, *K. pneumoniae*, *Salmonella* sp.	Blocks attachment; disrupts biofilms [168]
Mannose-based emulsion sensor	Diagnostic detection	Competitive displacement of mannan	*S. enterica*	Detection limit 100 cells/mL; 1 h assay [169]

**Table 9:** Representative sugar acid- and glucan-based Trojan Horse systems.

Platform	Function	Mechanism	Target	Key Outcome
ERNathG probe (glucuronic acid–naphthalimide)	Diagnostic probe	β-Glucuronidase-activated fluorescence	GUS-positive bacteria (*E. coli*, *S. warneri*)	Detection limit 1 CFU/mL; selective staining [178]
GLY-MSN (glucuronic acid–mesoporous silica nanoparticles)	Antibiotic delivery	Glucuronic acid-mediated bacterial targeting	*E. coli*	Enhanced antibacterial efficacy [179]
Glucan Lipid Particles (GLPs)	Antimycobacterial delivery	Macrophage recognition via glucan	*M. tuberculosis*	Targeted lung delivery; enhanced efficacy [180]
FLAPS (fluorescently labeled polysaccharides)	Metabolic imaging	Bacterial uptake via SusCD-like transporters	*Bacteroidetes*, gut microbiota	Real-time visualization of carbohydrate uptake [181]

**Table 10:** Representative β-glycoside-based Trojan Horse systems.

Platform	Function	Mechanism	Target	Key Outcome
β-Maltosyl thiosemicarbazone conjugates	Targeted antibiotic delivery	β-Glycoside recognition by bacterial PTS; intracellular hydrolysis	*C. difficile*, *B. subtilis*, *S. aureus*, MRSA	MIC 0.78–400 μg/mL; DNA gyrase inhibition [117]
β-Glycoside derivatives of 1-R-aminomethylphosphonic acid (l-AEP)	Targeted antibacterial agents	Bacterial β-glycosidase activation	*E. aerogenes*, *K. pneumoniae*, *S. enterica*	Selective activity against Gram-negative bacteria [187]
Salicin-based prodrugs (conceptual)	Antibiotic delivery	β-Glucosidase-triggered release	Gram-positive and Gram-negative pathogens	Proposed for future development

**Table 11:** Representative non-metabolizable sugar analog-based Trojan Horse systems.

Platform	Function	Mechanism	Target	Key Outcome
Azide-modified sugars (GalNAz, ManNAz, GlcNAz)	Fluorescent labeling	Metabolic incorporation into cell wall; click chemistry detection	*Bacteroides fragilis*, gut microbiota	Real-time anaerobic visualization; viability preserved [193]
FLAPS (fluorescently labeled polysaccharides)	Metabolic imaging	Bacterial uptake via SusCD-like transporters	Bacteroidetes, gut microbiota	Visualization of carbohydrate uptake dynamics [181]
^18^F-FDM, ^18^F-FSK	PET imaging	Maltodextrin transporter uptake; intracellular accumulation	*S. aureus* (including MRSA), *A. baumannii*	Specific infection imaging; distinguishes from inflammation [194]
2-Deoxyglucose, α-methylglucoside (conceptual)	Antibiotic delivery	Bacterial PTS/ABC transport; metabolic trapping	Various bacteria	Proposed for targeted delivery [189]
D-Allulose (conceptual)	Species-specific targeting	AlsE-dependent metabolism	Limited bacterial species	Potential for selective targeting [191]

**Table 12:** Representative GlcNAc- and GlcN-based Trojan Horse systems.

Platform	Function	Mechanism	Target	Key Outcome
Glucosamine–fluoroquinolone conjugates	Antibiotic delivery	Bacterial PTS-mediated uptake	*E. coli*, MRSA, *L. monocytogenes*, fungi	Enhanced activity; 100× lower cytotoxicity [206,207]
Glucosamine–lactate copolymers	Sustained antibiotic release	Surface glucosamine for bacterial binding	*M. smegmatis*	Rifampicin delivery for tuberculosis model [209]
GAC (GlcNAc) glycoconjugate vaccine	Immunization	GlcNAc as antigenic epitope	Group A *Streptococcus*	Elicits anti-GAC IgG; protective immunity [210,211]

**Table 13:** Representative MurNAc- and muropeptide-based Trojan Horse systems.

Platform	Function	Mechanism	Target	Key Outcome
GlcNAc–anhydroMurNAc–fluorophore conjugate	Cargo delivery	AmpG-mediated transport	*E. coli*	Confirms transporter accommodates bulky cargo [214]
UDP–MurNAc derivatives	Metabolic labeling	Incorporation into peptidoglycan biosynthesis	Various bacteria	Visualization of cell wall dynamics [215]

**Table 14:** Representative arabinose- and AXOS-based Trojan Horse systems.

Platform	Function	Mechanism	Target	Key Outcome
Juglone–arabinose conjugates	Antibiotic delivery	Arabinose-mediated bacterial uptake	Various bacteria	Antimicrobial activity comparable to vancomycin/gentamicin [218]
C2-linked D-arabinofuranose–polystyrene microbeads	Targeted binding + biofilm disruption	Specific recognition of bacterial surface arabinose	*S. aureus*	Selective binding; biofilm disruption [219]
AXOSs (arabinoxylan-oligosaccharides)	Cargo delivery (conceptual)	ABC transporter (BIAXBP) uptake	Gut bacteria	Proposed for targeted delivery to intestinal microbiota [220,221]

**Table 16:** Representative vitamin-based Trojan Horse systems.

Vitamin	Platform	Function	Target	Key Outcome
B12	B12–ampicillin conjugate	*Antibiotic delivery*	*E. coli*, *S. typhimurium*	500× and 60× enhanced activity [243]
B12	B12–chloramphenicol conjugate	*Antibiotic delivery*	*E. coli*	Maintained efficacy; reduced toxicity [243]
B12	B12–PNA/oligonucleotide conjugates	*Antisense gene silencing*	*E. coli*, *S. typhimurium*	Sequence-specific bacterial killing [244,245]
B12	PMAG–DFOA–CT–B12 (multifunctional)	Oral/intravenous delivery	*P. aeruginosa*	Enables multiple administration routes [246]
B2 (riboflavin)	VanB2 (riboflavin–vancomycin)	Photodynamic therapy + antibiotic release	MRSA, VRE	Dual action: ROS generation + vancomycin release [247]
B2	Riboflavin–iron oxide nanozymes	Wound healing	Oral bacteria	Enhanced healing; antimicrobial activity [248]
C (ascorbic acid)	Vitamin C alone	Oxidative stress + efflux inhibition	CR-hvKP	Bactericidal; anti-biofilm; anti-virulence [249]
Folic acid	Folic acid–CeO_2_ nanoparticles	Targeted antimicrobial	MRSA	>95.6% growth inhibition [250]
Folic acid	Folic acid–Ag nanoparticles	Targeted delivery	Various bacteria	Enhanced antibacterial activity [251]
Multiple	Vitamin–metallic nanoparticles (Au, Ag, Fe_3_O_4_)	Receptor-mediated uptake + cytotoxic payload	Various bacteria, cancer cells	Improved selectivity; reduced toxicity [252]

**Table 17:** Representative lectin-based Trojan Horse systems.

Platform	Function	Mechanism	Target	Key Outcome
LecA/LecB-targeted prodrugs	Self-destructive antibiotic release	Bacterial lectin recognition triggers drug activation	*P. aeruginosa* biofilms	Localized antibiotic release; reduced toxicity [283,284]
Apoferritin–WGA–ampicillin	Targeted antibiotic delivery	WGA binding to bacterial surface GlcNAc	*B. subtilis*	10× enhanced activity; effective in blood [285]
WGA/LCA lectins	Intracellular targeting	Lectin accumulation in bacterial hideouts	UPEC (intracellular)	Potential for delivery to bacterial reservoirs [286]
ConA-modified micro-rockets	Active bacterial capture + drug delivery	ConA binding to bacterial surface mannose	*E. coli*	Real-time capture; simultaneous drug carrier delivery [287]

**Table 18:** Representative antibody-based Trojan Horse systems.

Platform	Payload	Target	Key Outcome
DSTA4637A (anti-teichoic acid–rifamycin)	Rifamycin-class antibiotic	*S. aureus* (including MRSA)	7.6 log_10_ CFU reduction; 14-day persistence; favorable PK/PD [22,291]
Anti-LPS 26F8–G2637	Arylamycin antibiotic	*P. aeruginosa*	Intracellular killing at 100× lower concentration than free drug [292]
PIAS (antibody–IRDye 700DX)	Photosensitizer	MRSA, fungi, viruses	Near-infrared activated; physical membrane disruption [25]
Streptavidin–eosin–IgG	Photosensitizer (eosin)	*S. aureus*	8-log CFU reduction upon green light irradiation [26]
Anti-*S. aureus*–M13 phage–Alexa Fluor 750	Fluorescent dye	*S. aureus*	3.7× enhanced fluorescence signal; in vivo imaging [293]

**Table 19:** Representative engineered phage-based Trojan Horse systems.

Platform	Payload	Mechanism	Target	Key Outcome
T7 phage syringes	Penicillin G	Spontaneous encapsulation; injection into cytoplasm	*E. coli* (penicillin-resistant)	Overcomes β-lactamase resistance [297]
M13 phage–neomycin–chloramphenicol	Chloramphenicol (up to 40,000 molecules/phage)	Aminoglycoside branched linker; ZZ domain targeting	*S. aureus*, *S. pyogenes*, *E. coli*	20,000× enhanced efficacy; reduced immunogenicity [298,299]
M13 phage–prodrug (chloramphenicol ester)	Chloramphenicol (prodrug)	Labile ester bond; antibody-mediated targeting	*S. aureus*	10–20× local concentration effect; prodrug activation [300]
M13 phage–fluorophore (Alexa Fluor 750)	Fluorescent dye	Covalent attachment to coat proteins	F-pili-expressing *E. coli*; antibody-targeted *S. aureus*	3.7× enhanced in vivo imaging signal [293]

**Table 20:** Representative phage endolysin-based Trojan Horse systems.

Platform	Function	Mechanism	Target	Key Outcome
Lysep3-D8 (domain shuffling)	Antimicrobial	CBD disrupts LPS; catalytic domain degrades peptidoglycan	*E. coli*, *P. aeruginosa*, *A. baumannii*	Expanded host range; kills Gram-negative bacteria [24]
Innolysin Ec21 (RBP–endolysin)	Antimicrobial	Pb5 binds FhuA; endolysin degrades peptidoglycan	*E. coli* (cephalosporin-resistant)	Activity against resistant strains [305]
Lysocin (pyocin S2–GN4)	Antimicrobial	Ton-dependent import; periplasmic peptidoglycan degradation	*P. aeruginosa*	Serum stability; biofilm disruption; in vivo protection [306]
ClyS, ClyF (chimeric lysins)	Antimicrobial	Domain shuffling for enhanced activity	MRSA, biofilms	Improved stability; activity in complex media [307,308]
CBD–GFP fusions	Diagnostic	CBD-mediated binding; fluorescent detection	*Listeria* spp., *Clostridium* spp.	Multiplex serovar differentiation; in situ detection [309,310]
CBD3–IRDye 700DX	Photodynamic therapy	CBD targeting; ROS generation upon light activation	*S. aureus*, MRSA, biofilms, intracellular bacteria	Biofilm disruption; intracellular killing [27]

**Table 21:** Representative bacterial enzyme-activated prodrug systems.

Enzyme	Prodrug Platform	Active Agent	Target	Key Outcome
β-Lactamase	Cephalosporin–ciprofloxacin conjugate	Ciprofloxacin	β-Lactamase-producing *E. coli*	Selective killing of resistant strains; preserves microbiota [35]
Nitroreductase (NTR)	2-Nitrothiazole ester–moxifloxacin	Moxifloxacin	*M. tuberculosis* persisters	Kills dormant subpopulations [313]
Mycobacterial nitroreductases	Pretomanid, delamanid	Reactive metabolites	*M. tuberculosis*	Selective activation in mycobacteria [314]
Nitroreductase (NfsB)	Piperazine diazeniumdiolate (nitroaromatic)	Nitric oxide (NO)	*E. coli*	Up to 94% viability reduction [315]
Glyoxalase II (GloB)	POM-ERJ (pivaloyloxymethyl ester)	DXR inhibitor (isoprenoid biosynthesis)	*S. aureus*, MRSA	10–50× resistance in GloB mutants [316]
Mycobacterial esterases	Benzoate ester prodrugs	Active benzoate derivatives	Mycobacteria	Selective activation vs. mammalian hydrolases [317]

**Table 23:** Comparative analysis of Trojan Horse platforms for antibacterial therapy.

Parameter	Metabolite-Based Mimics	Surface Receptor Targeting (Antibodies/Lectins)	Bacteriophage-Based Systems	Extracellular Vesicles	Enzyme-Activated Prodrugs
Mechanism of action	Active transport via nutrient uptake systems (siderophores, sugars, vitamins)	Binding to surface antigens or carbohydrates; often followed by host cell internalization	Phage-mediated injection of payload; endolysin activity; surface display	Fusion with target membranes; delivery of packaged cargo	Bacterial enzyme converts inactive prodrug to active antibiotic
Targeting specificity	High (depends on transporter conservativeness)	Very high (antibody–antigen recognition)	Extremely high (evolutionarily refined host recognition)	Moderate to high (intrinsic tropism for parent species)	Moderate to high (depends on enzyme specificity)
Cargo capacity	Low (small molecules, ~1–2 payloads per carrier)	Low to moderate (antibody: 2–8 drugs; lectin: variable)	Very high (up to 40,000 molecules per phage)	High (naturally encapsulated diverse cargo)	N/A
Immunogenicity	Low (small molecules, often endogenous)	Moderate to high (protein-based; can be humanized)	Moderate (reduced by chemical modification)	Moderate to high (bacterial origin)	Low (small molecules)
Manufacturing complexity	Low (standard chemical synthesis)	High (recombinant production, conjugation)	Moderate (phage propagation, chemical modification)	High (isolation, loading, characterization)	Low to moderate (chemical synthesis)
Clinical status	FDA-approved (cefiderocol); several in clinical trials	Preclinical; AACs in early trials	Preclinical; phage therapy approved in some countries	Preclinical	Several approved for clinic (e.g., pretomanid for TB)
Primary advantage	Simple design, well-understood uptake pathways	Unmatched specificity; access to intracellular reservoirs	High cargo capacity; programmable payloads	Natural nanocarriers; cross biological barriers	Simple design
Primary limitation	Resistance development via transporter mutations	Immunogenicity; high cost	Regulatory hurdles; potential horizontal gene transfer	Heterogeneity; scale-up challenges	Requires expressed enzyme in particular bacterial specie

N/A—not applicable.

## Data Availability

Data sharing not applicable. No new data were created or analyzed in this study.

## Abbreviations

The following abbreviations are used in this manuscript:

[^18^F]FDM	α-1,4-linked maltose
[^18^F]FSK	α-1,3-linked sakebiose
AACs	antibody–antibiotic conjugates
ABC	ATP-binding cassette
ADME	absorption, distribution, metabolism, and elimination
AG	arabinogalactan
AIE	aggregation-induced emission
AMPs	antimicrobial peptides
AMSs	azide-modified sugars
ATP	adenosine triphosphate
AXOSs	arabinoxylan-oligosaccharides
BCG	*Bacille Calmette-Guérin*
BEVs	bacterial extracellular vesicles
BODIPY	boron dipyrromethene/4,4-difluoro-4-bora-3a,4a-diaza-s-indacene
BP	binding protein
BTS	bacterial transport system
CBD	cell binding domain
CDG-Tre	cephalosphorinase-dependent green trehalose
CEV	extracellular vesicles with ceftiofur
CEVH	extracellular vesicles with ceftiofur in hydrogel
CFU	colony-forming unit
ConA	concanavalin A
CPPs	cell-penetrating peptides
DMN-Tre	4-N,N-Dimethylamino-1,8-naphthalimide–trehalose conjugate
Dpp	dipeptide permease
ERNathG	β-d-glucuronic acid 4-hydroxy-1,8-naphthalimide p-toluene sulfonyl
ESBL	extended-spectrum beta-lactamase
EVs	extracellular vesicles
FDA	US Food and Drug Administration
FLAPSs	fluorescently labeled polysaccharides
GAC	group A carbohydrate
GlcN	glucosamine
GlcNAc	N-acetylglucosamine
GLPs	glucan lipid particles
GLUT	glucose transporter
GLY-MSNs	glycosylated mesoporous silica nanoparticles
g-MVs	gentamicin-induced vesicles
GUS	bacterial β-glucuronidase
LacAC4A	lactose-modified azo-calix [4]arene
L-AEP	L-R-aminoethylphosphonic acid
LCA	Lens culinaris agglutinin
LPS	lipopolysaccharide
mAGP	mycolyl–arabinogalactan–peptidoglycan
Mal-Cz	maltose–carbazole
MDNPs	maltodextrin nanoparticles
MDR	multidrug-resistant
MIC	minimum inhibitory concentration
MMCC	mannose-maltose-colistin conjugate
MOFs	metal–organic frameworks
MP-MENP	mannose-functionalized manganese-eumelanin coordination nanoparticles
MRSA	methicillin-resistant *Staphylococcus aureus*
MurNAc	N-acetylmuramic acid
NA	nalidixic acid
NCC	nanocrystalline cellulose
NIS	nonribosomal independent synthesis
n-MVs	naturally released membrane vesicles
NO	nitric oxide
NRPS	nonribosomal peptide synthetase
NTR	nitroreductase
Opp	oligopeptide permease
PAMPs	pathogen-associated molecular patterns
PA	polyamine
PDI	photodynamic inactivation
PEG	polyethylene glycol
PEP	phosphoenolpyruvate
PET	positron emission tomography
PG	peptidoglycan
PIAS	Photoimmuno-Antimicrobial Strategy
Pks13	polyketide synthase 13
PLGA	poly(lactic-co-glycolic acid)
PNA	peptide nucleic acid
POM	pivaloyloxymethyl
PS	photosensitizer
PTS	phosphotransferase system
ROS	reactive oxygen species
SBP	substrate-binding protein
SGLT1	sodium–glucose cotransporter 1
SpEV	*Streptococcus parauberis* extracellular vesicles
TCC2Tre	tricarbocyanine trehalose conjugate
TDD	Targeted drug delivery
THS	“Trojan Horse” Strategy
TMG	Methyl-β-D-thiogalactopyranoside
TM-TMP	thiomaltose trimethoprim conjugate
Tre-Cz	trehalose–carbazole
UPEC	uropathogenic *E. coli*
WGA	wheat germ agglutinin
